# A systematic review and evaluation framework for IoB-based adaptive health coaching systems

**DOI:** 10.3389/fdgth.2026.1816418

**Published:** 2026-08-21

**Authors:** P. W. C. Prasad, Daniel Patricko Hutabarat, Md Shohel Sayeed, Golam Md Mohiuddin

**Affiliations:** 1 International School, Duy Tan University, Danang, Vietnam; 2 Department of Computer Engineering, Faculty of Engineering, Bina Nusantara University, Jakarta, Indonesia; 3 Centre for Intelligent Cloud Computing, CoE for Advanced Cloud, Faculty of Information Science and Technology, Multimedia University, Melaka, Malaysia; 4 Faculty of Information Science and Technology, Multimedia University, Melaka, Malaysia

**Keywords:** behavioural analytics, edge computing, eldercare, health coaching, IoB, personalization, wearables

## Abstract

**Introduction:**

The convergence of the Internet of Things (IoT), artificial intelligence (AI), and behavioural analytics has contributed to the emergence of the Internet of Behaviours (IoB) as a framework for personalized and context-aware digital health interventions. This study systematically reviews and synthesizes the architectures, sensing technologies, behavioural analytics methods, machine learning approaches, intervention strategies, and evaluation practices underlying IoB-enabled adaptive health coaching systems.

**Methods:**

Following the PRISMA 2020 guidelines, a systematic literature review was conducted across IEEE Xplore, Scopus, Web of Science, PubMed, and ACM Digital Library. A total of 75 eligible peer-reviewed studies published between 2015 and 2025 met the predefined eligibility criteria and were included in the final evidence synthesis.

**Results:**

The synthesis identified wearable and multimodal sensing, edge cloud architectures, AI-driven behavioural modelling, and just-in-time adaptive interventions (JITAIs) as recurring technological and intervention components. Comparatively stronger empirical support was observed for continuous monitoring, behavioural and physiological pattern recognition, remote patient monitoring, elderly care, and selected chronic disease applications. In contrast, evidence for advanced adaptive coaching, digital twins, metaverse-enabled healthcare, behavioural simulation, and emerging AI or large language model (LLM) enabled systems was more heterogeneous and frequently derived from prototype, simulation-based, or early-stage evaluations. Across the evidence base, substantial variation was observed in study designs, populations, datasets, intervention types, validation settings, and outcome measures, limiting direct cross-study comparison and conclusions regarding comparative effectiveness. Key limitations included insufficient longitudinal and clinical validation, incomplete reproducibility, fragmented interoperability, heterogeneous evaluation metrics, and unresolved privacy and governance challenges.

**Discussion:**

Based on recurring architectural, technological, and methodological patterns identified across the included studies, this review proposes a synthesized reference architecture and evaluation framework for IoB enabled adaptive health coaching. These evidence-informed conceptual synthesis frameworks are intended to guide future research and implementation rather than serve as universally validated standards. Future research should prioritize longitudinal and multi-site validation, representative datasets, reproducible reporting, interoperable clinical integration, privacy-preserving analytics, and the combined evaluation of technical, behavioural, and clinically meaningful outcomes.

## 1 Introduction

The rapid convergence of the Internet of Things (IoT), artificial intelligence (AI), and behavioural analytics has led to the emergence of the Internet of Behaviours (IoB), a paradigm that focuses on capturing, analysing, and influencing human behaviours based on digital interaction and sensor data. IoB systems enable adaptive health coaching by leveraging multimodal data from wearables, smart devices, and environmental sensors to provide personalized recommendations that encourage healthier lifestyles, improve clinical outcomes, and enhance self-management of chronic conditions (1–3). The proliferation of wearable health devices and sensor-driven ecosystems has fundamentally transformed healthcare delivery, moving it from episodic and reactive models to continuous, personalized, and preventive care (4, 5). Adaptive health coaching systems powered by IoB collect physiological, behavioural, and contextual data to identify risk factors, predict adverse health events, and provide real-time interventions. For example, smartwatches, glucose monitors, and environmental IoT nodes work together to track activity levels, heart rate variability, sleep quality, and diet compliance (6, 7). These insights inform personalized behavioural nudges, medication reminders, and activity coaching aligned with behaviour change theories and health informatics frameworks (8).

However, deploying IoB systems for health coaching faces challenges of privacy, explainability, and scalability. Privacy concerns stem from the sensitive nature of behavioural and health data, which often require federated learning or blockchain-based protection to minimize exposure (9, 10). Explainable AI techniques within IoB allow healthcare professionals and users to understand the rationale behind recommendations, thus improving trust and adoption (11, 12). Scalability challenges arise when moving from pilot deployments to real-world, multi-user environments, especially for eldercare and chronic disease management applications (13, 14). The purpose of this research is to provide a comprehensive literature synthesis on IoB-based adaptive health coaching systems, exploring their architectures, sensing technologies, machine learning approaches, behavioural analytics, and clinical applications. This study systematically reviews IoB implementations across domains such as elderly care, chronic disease management, mental health support, physical activity coaching, and nutrition monitoring, while addressing critical factors including interoperability, user experience, and data governance (2, 15, 16).

### 1.1 Evolution of IoB in healthcare

The concept of IoB emerged as an extension of IoT, integrating behavioural analytics to understand and influence user actions (17). While IoT focused primarily on device connectivity and data collection, IoB emphasizes actionable insights and behaviour change through data-driven interventions. Applications in healthcare and wellness are among the most prominent drivers of IoB adoption, ranging from preventive care to clinical decision support (18, 19). IoB has gained attention in both academic research and industry applications. Recent studies highlight its transformative role in eldercare, where robot-assisted systems and smart home environments improve safety, independence, and overall quality of life (7, 20). Similarly, adaptive digital coaching platforms leverage IoB to enhance patient adherence, disease self-management, and rehabilitation programs (2, 21). Integration with behaviour change techniques, such as goal setting, feedback loops, and gamification, has proven effective in sustaining long-term engagement (22, 23). Furthermore, IoB is multi-domain, influencing areas like food safety monitoring, smart supply chains, and public health planning, which indirectly support health outcomes (1, 16). This cross-domain versatility positions IoB as a cornerstone for adaptive health coaching, as behavioural data often spans multiple life contexts beyond clinical encounters.

### 1.2 Rationale for adaptive health coaching systems

Traditional healthcare models rely on retrospective interventions, where treatment decisions occur after symptom onset. Adaptive health coaching flips this model by providing real-time, personalized interventions based on predictive behavioural analytics. By continuously monitoring user-specific metrics such as activity, nutrition, emotional state, and physiological patterns, IoB-driven systems can detect deviations from healthy norms and prompt timely corrective actions (6, 24).

The rationale for integrating IoB in health coaching includes:

- Early Detection and Intervention: Systems can pre-emptively identify high-risk conditions like COPD exacerbations, glucose fluctuations, or fall risks (25, 26).
- Behavioural Personalization: By fusing multi-modal data, coaching strategies are tailored to individual routines, motivations, and constraints, improving adherence (22, 27).
- Scalability of Care: IoB systems reduce reliance on continuous human supervision, allowing digital platforms and edge AI to support large user populations (28, 29).

Moreover, the integration of explainable AI ensures that recommendations are transparent and clinically interpretable, which is essential for user trust and regulatory compliance (9, 11).

### 1.3 Challenges and research gaps

Despite its promise, IoB-based adaptive health coaching faces several research challenges:

- Data Privacy and Security: Sensitive health and behaviour data require multi-layered security, often supported by blockchain and federated learning to avoid centralized exposure (10, 30).
- Interoperability and Standardization: Disparate IoT devices, wearables, and health record systems often lack uniform communication protocols (8, 31).
- Behavioural Variability: Human behaviour is inherently nonlinear and context-dependent, demanding adaptive algorithms that can generalize without excessive false alerts (32, 33).
- Clinical Validation: Many IoB implementations remain in pilot stages or simulated environments, necessitating longitudinal trials for robust clinical validation (14, 21).

Addressing these gaps requires multi-disciplinary approaches, layered IoT architectures, and data governance frameworks that balance innovation with ethical responsibility (34, 35).

## 2 Review methodology

This study adopts a structured systematic review methodology to identify, evaluate, and synthesize literature on Internet of Behaviours (IoB)-enabled adaptive health coaching systems. The review methodology follows the Preferred Reporting Items for Systematic Reviews and Meta-Analyses (36) guidelines to ensure transparency, reproducibility, and methodological rigor in the identification, screening, and synthesis of relevant studies. The PRISMA framework guided the literature search strategy, study selection process, and reporting structure of this review.

### 2.1 Databases and information sources

A comprehensive literature search was conducted across five major academic databases: IEEE Xplore, Scopus, Web of Science, PubMed, and ACM Digital Library, to ensure broad coverage of peer-reviewed research related to healthcare informatics, artificial intelligence, the Internet of Things (IoT), and interdisciplinary digital health domains. These databases were selected due to their extensive indexing of high-quality journal articles and conference proceedings relevant to Internet of Behaviours (IoB)-enabled adaptive health coaching systems. The review focused on studies published between January 2015 and December 2025, reflecting the period during which IoB and behaviour-driven healthcare technologies gained significant academic and practical attention. The final database search was conducted on 15 December 2025 to ensure inclusion of the most recent eligible studies. Searches were performed within the Title, Abstract, and Author Keywords fields, where supported by the respective database interfaces. Only peer-reviewed journal articles and conference papers published in English and meeting the predefined eligibility criteria were considered for inclusion. To enhance comprehensiveness, backward and forward citation screening was performed on the included studies to identify additional relevant publications. Supplementary sources, including book chapters, technical reports, and preprints, were consulted only to provide contextual background on emerging topics such as federated learning, explainable artificial intelligence (XAI), conversational agents, and IoB architectures. However, these sources were excluded from the formal study selection, quality assessment, data extraction, and evidence synthesis processes to maintain methodological rigor and consistency.

**Figure 1:**
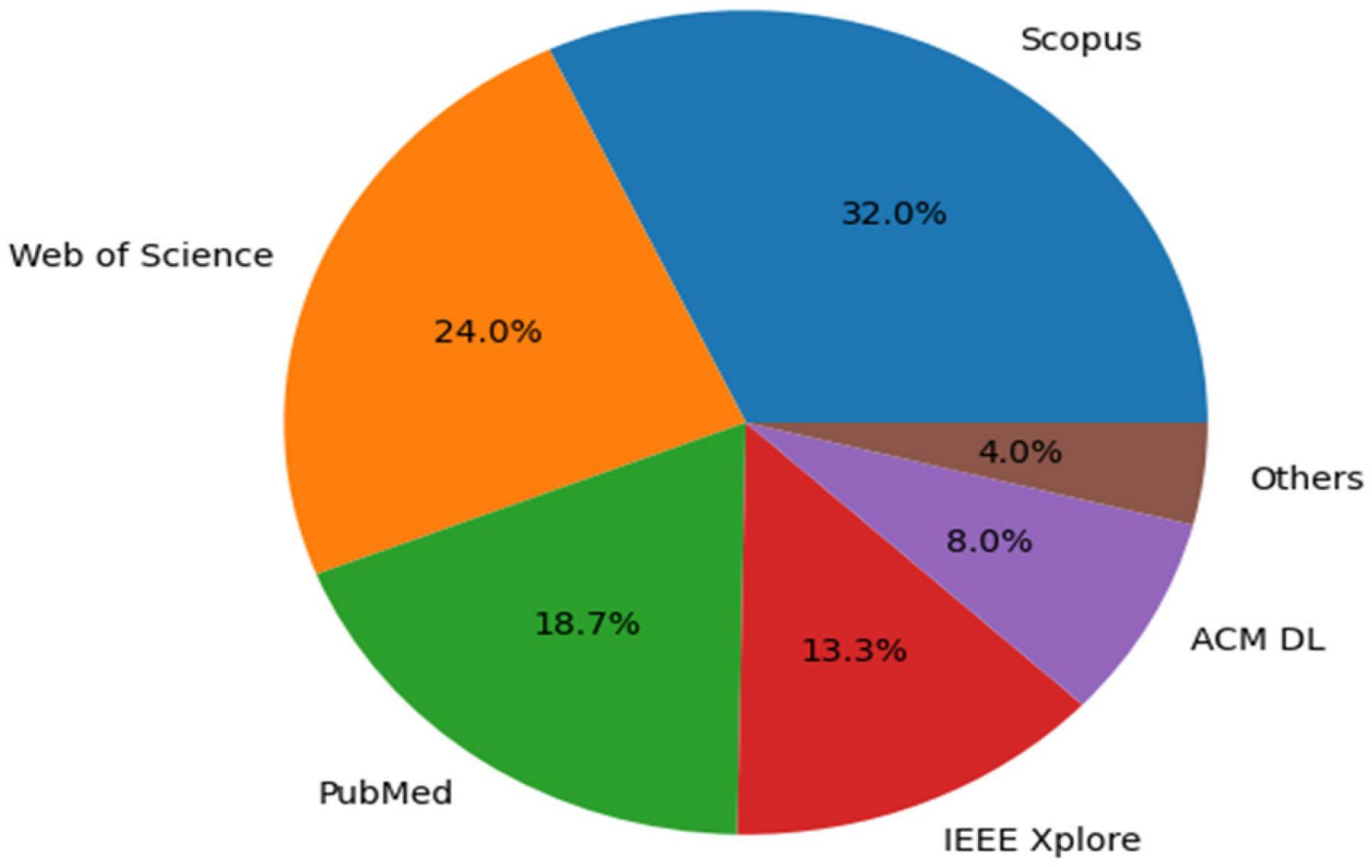
Percentage distribution of included studies across major databases and additional sources.

As illustrated in Figure 1, the distribution of included studies across the selected databases reflects a comprehensive and balanced search strategy. Scopus contributed the largest proportion of the included studies (32.0%), followed by Web of Science (24.0%) and PubMed (18.7%), demonstrating strong representation from both healthcare and interdisciplinary research domains. IEEE Xplore accounted for 13.3% of the included literature, providing substantial coverage of IoT architectures, sensor technologies, and intelligent healthcare systems, while the ACM Digital Library contributed 8.0%, reflecting its relevance to computing and data-driven healthcare research. The remaining 4.0% of studies were identified through supplementary sources, including backward and forward citation screening. This diversified search strategy ensured broad coverage of technological, clinical, behavioural, and methodological perspectives relevant to IoB-enabled adaptive health coaching systems.

### 2.2 Research question and PICOS framework

To clearly define the scope of the review, the research question was formulated using the PICOS (Population, Intervention, Comparator, Outcome, and Study Design) framework. The research question guiding this study is: “How are Internet of Behaviours (IoB) technologies implemented in adaptive health coaching systems, and what architectures, sensing technologies, machine learning methods, and evaluation frameworks are used to support behaviour-driven healthcare interventions?” The PICOS components used to define the review scope, study selection criteria, and research objectives are summarized in Table 1.

**Table 1:** PICOS criteria for study selection and review scope.

PICOS Element	Description
Population (P)	Individuals or patient groups monitored using Internet of Things (IoT) or Internet of Behaviours (IoB) enabled healthcare systems, including patients with chronic conditions, elderly individuals, or users of wearable health monitoring technologies.
Intervention (I)	IoB-based adaptive health coaching systems that utilize wearable sensors, IoT devices, artificial intelligence models, and behavioural analytics to deliver personalized health interventions.
Comparator (C)	Traditional healthcare monitoring systems, conventional digital health applications, or non-adaptive health monitoring technologies without behaviour-driven intelligence.
Outcome (O)	Behavioural change, improved health outcomes, increased adherence to treatment or lifestyle interventions, and enhanced system performance in health monitoring and coaching applications.
Study Design (S)	Primary empirical studies, including experimental research, pilot implementations, clinical evaluations, observational studies, and real-world healthcare implementations related to IoB-enabled adaptive health coaching systems.

### 2.3 Search strategy and keywords

A structured Boolean search strategy was developed to systematically identify literature at the intersection of Internet of Behaviours (IoB), adaptive health coaching, and IoT-enabled healthcare systems. The search queries were designed to capture studies integrating behavioural data analytics, intelligent intervention mechanisms, and connected health technologies. Although minor syntactic adjustments were made to accommodate database-specific indexing rules, the core search structure followed the format:

(“Internet of Behaviours” OR “IoB”) AND (“health coaching” OR “adaptive intervention” OR “personalized intervention” OR “Just-in-Time Adaptive Intervention” OR “JITAI” OR “behavioural analytics”) AND (“IoT healthcare” OR “wearables” OR “digital health” OR “remote patient monitoring”)

To ensure comprehensive coverage, additional complementary keyword combinations were employed, including terms such as “behaviour-driven IoT,” “adaptive health systems,” “digital therapeutics,” “edge AI in healthcare,” and “federated learning in health IoT.” These supplementary searches were particularly useful in capturing emerging interdisciplinary research where IoB terminology was not explicitly used but conceptually aligned with behaviour-aware intelligent health systems. Where database functionality permitted, search filters were applied to restrict results to peer-reviewed journal articles and conference proceedings published in English. Additional records identified through backward and forward reference screening were subsequently evaluated against the same eligibility criteria to ensure consistency throughout the study selection process. The search strategy prioritized relevance to healthcare and behaviour-driven intelligent systems while maintaining sensitivity to novel architectures, privacy-preserving frameworks, and AI-enabled adaptive interventions. The complete database-specific search strategies, including exact search strings, database-specific syntax adaptations, search fields, filters, publication-type limits, language restrictions, search dates, and records retrieved from each database, are provided in Supplementary Table S1 (provided as Supplementary Material 2) to facilitate independent replication of the search process.

### 2.4 Inclusion criteria

Studies were considered eligible for inclusion if they met all of the following criteria. First, the publication had to be a peer-reviewed journal article or conference paper reporting primary empirical research to ensure academic rigor, methodological reliability, and relevance to the review objectives. Second, the study must have been published between January 2015 and December 2025, reflecting the period during which Internet of Behaviours (IoB) and behaviour-driven IoT healthcare systems gained significant research attention. Third, only publications written in English were included to maintain consistency in interpretation and analysis. In terms of thematic relevance, studies were required to focus on IoB, behaviour-driven Internet of Things (IoT) systems, or adaptive digital health coaching frameworks. Eligible studies had to incorporate at least one of the following core components:

- Behaviour monitoring using IoT devices or wearable sensors
- Adaptive, personalized, or Just-in-Time interventions targeting health behaviour change
- Machine learning or artificial intelligence–driven decision-support systems for behaviour-based healthcare applications; or
- integrated architectures combining sensing, data analytics, and intervention mechanisms within healthcare contexts.

Eligible studies were limited to primary empirical research, including experimental validation studies, pilot implementations, clinical evaluations, observational studies, and real-world healthcare deployments relevant to IoB-enabled adaptive health coaching systems. Review articles, survey papers, systematic reviews, and scoping reviews were consulted to provide contextual background and identify additional references; however, they were excluded from the formal study selection, quality assessment, data extraction, and evidence synthesis processes. To maintain alignment with the review objectives, the evidence synthesis primarily focused on healthcare-related IoB applications, including adaptive health coaching, chronic disease management, elderly care, mental health support, wearable health monitoring, and remote patient monitoring. Non-healthcare studies were considered only when they provided transferable architectural, behavioural modelling, sensing, or intervention mechanisms directly applicable to healthcare contexts.

### 2.5 Exclusion criteria

Studies were excluded from the review if they met one or more of the following criteria. First, non-peer-reviewed materials including blogs, opinion pieces, white papers, marketing reports, magazine articles, informal web content, preprints, book chapters, and technical reports were excluded from the formal review process to maintain academic rigor and methodological reliability. In addition, review articles, survey papers, systematic reviews, and scoping reviews were excluded from the formal study selection and evidence synthesis processes, although they were consulted for background context and reference identification where relevant. Second, publications presenting purely conceptual or speculative discussions without substantive technical, architectural, empirical, or evaluative components were omitted, as the review focused on implementable and analytically grounded IoB-based health systems. Third, studies unrelated to healthcare applications were excluded unless they offered directly transferable architectural, behavioural modelling, or adaptive intervention frameworks relevant to IoB-enabled health coaching contexts. Fourth, publications addressing general IoT infrastructure, networking protocols, or hardware systems without incorporating behavioural modelling, adaptive feedback mechanisms, or AI-driven health interventions were not considered eligible. Finally, duplicate records identified across databases were removed during the screening process to prevent redundancy and ensure analytical consistency. Media articles, encyclopaedia entries, and other non-academic sources were systematically excluded to preserve the credibility, transparency, and scholarly integrity of the review.

### 2.6 Screening and selection process

**Figure 2:**
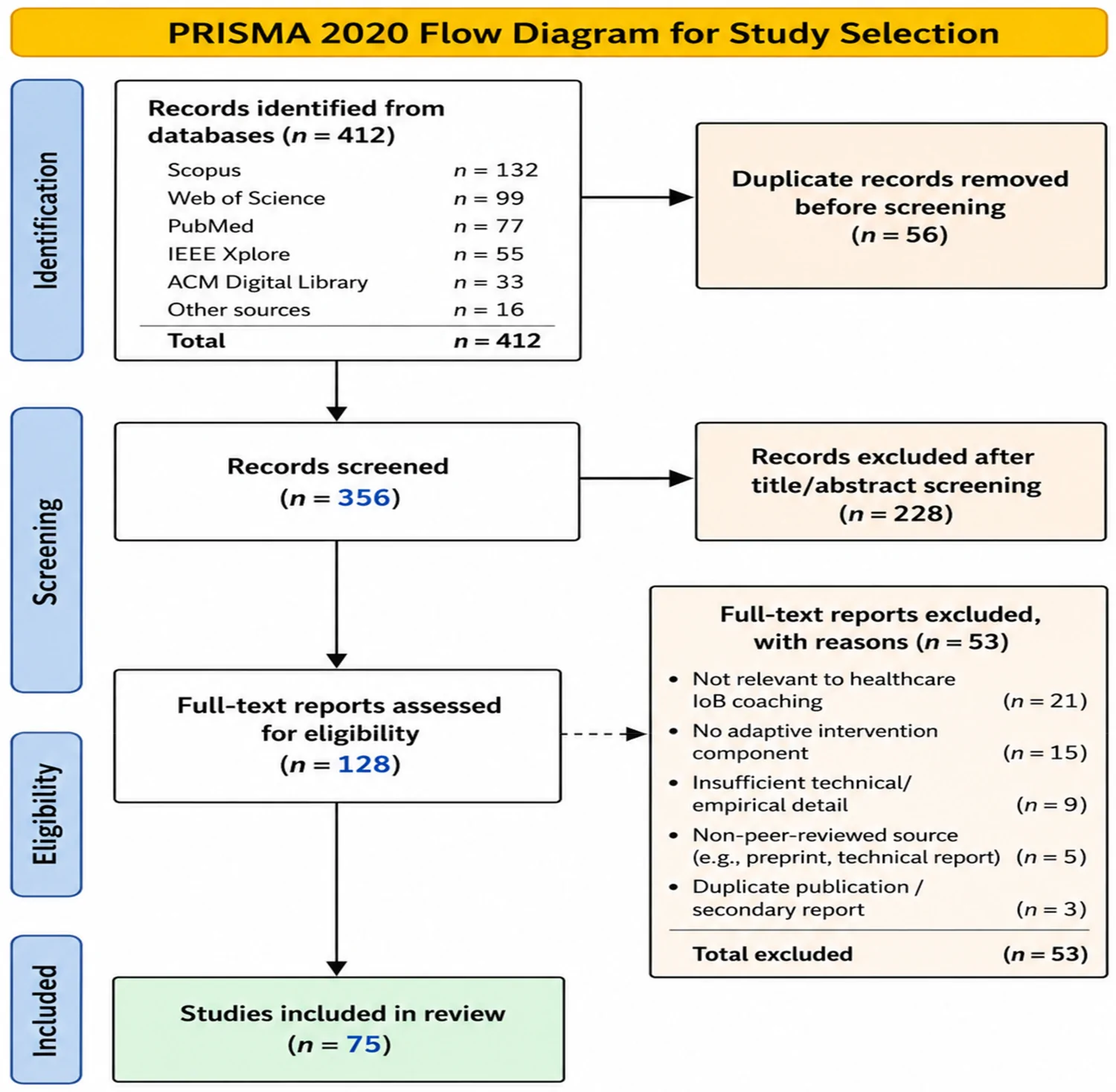
PRISMA flow diagram illustrating the study screening and selection process.

The literature search across the five selected databases yielded a total of 412 records. After removing duplicate entries (*n* = 56), 356 unique records remained for screening. The study selection process was conducted in accordance with the PRISMA 2020 guidelines and involved two independent reviewers (PWCP and DPH) to ensure methodological rigor, transparency, and consistency throughout the review process. The screening procedure consisted of three sequential stages. In the first stage, the titles and abstracts of all 356 records were independently assessed against the predefined inclusion and exclusion criteria. Studies that were clearly unrelated to Internet of Behaviours (IoB), adaptive health coaching, behavioural analytics, or healthcare-related IoT applications were excluded. Following this stage, 228 records were removed due to insufficient thematic relevance, resulting in 128 studies being retained for full-text assessment. In the second stage, the full-text versions of the 128 shortlisted studies were independently evaluated by both reviewers. The assessment focused on the presence of key characteristics relevant to the review scope, including behavioural monitoring mechanisms, adaptive or personalized intervention strategies, artificial intelligence-driven decision support, and integrated sensing analytics actuation architectures within healthcare contexts. During this stage, 53 studies were excluded because they failed to satisfy one or more eligibility criteria, including limited healthcare relevance, absence of adaptive intervention or behavioural modelling components, insufficient empirical validation, inadequate methodological reporting, or classification as review articles, survey papers, preprints, technical reports, or book chapters. Where multiple exclusion criteria applied to a study, the primary reason for exclusion was recorded during the full-text assessment process. A detailed breakdown of study selection and exclusion decisions is provided in the PRISMA 2020 flow diagram Figure 2. Any disagreements arising during either the title and abstract screening stage or the full-text assessment stage were resolved through discussion and consensus between the two reviewers. Where consensus could not be reached, a third senior reviewer (MSS) was consulted to make the final inclusion decision. Following the full-text review process, 75 studies satisfied all eligibility criteria and were included in the final synthesis. These studies span multiple healthcare application domains, including chronic disease management, medication adherence monitoring, elderly care, mental health support, wearable-based adaptive coaching systems, and privacy-preserving IoB-enabled healthcare frameworks.

To clearly distinguish the formal evidence base from references used solely for background or contextual purposes, the 75 studies included in the final evidence synthesis are listed in Supplementary Table S2 (provided as Supplementary Material 3). For each included study, the table summarizes publication type, study design, healthcare domain, population or dataset, IoB, IoT components, AI or behavioural analytics methods, evaluation setting, and key outcomes. References not listed in Supplementary Table S2 were excluded from the formal evidence synthesis and were used only, where relevant, to provide background context, support methodological framing, or discuss emerging research directions. The included studies constitute the evidentiary basis for the taxonomy development, architectural synthesis, and evaluation framework proposed in this review. Figure 2 presents the PRISMA 2020 flow diagram illustrating the identification, screening, eligibility assessment, and final study selection process.

### 2.7 Data extraction and synthesis approach

A structured data extraction framework was developed to systematically capture and compare key attributes from each of the 75 included studies. Study-level characteristics and findings are reported in Supplementary Table S2, including publication type, study design, healthcare domain, population or dataset, IoB/IoT components, AI or behavioural analytics methods, evaluation setting, and key outcomes. Additional information was extracted regarding data modalities employed, detection methodologies, machine learning techniques, architectural design, evaluation metrics, and implemented privacy or governance mechanisms. The extracted data were synthesized into structured analytical outputs, forming the basis of Table 4 (functional synthesis of IoB-enabled adaptive health coaching systems), Table 5 (comparative synthesis of AI and IoB technology approaches), and Table 6 (comparative synthesis of healthcare application domains). Detailed mappings of evaluation metrics, dataset characteristics, and hardware software integration variables were retained as supplementary materials to support transparency and traceability while maintaining a focused comparative synthesis in the main text. A comparative narrative and thematic synthesis approach was employed to identify cross-cutting themes, architectural patterns, methodological trends, differences in evidence maturity, and persistent research gaps across the included studies. In addition, a thematic synthesis approach was applied to group recurring architectural, technological, and behavioural patterns identified throughout the literature. The resulting categories and framework components were derived by consolidating conceptually similar elements into higher-level synthesis structures, thereby facilitating comparative analysis and the development of the proposed reference models. The proposed reference architecture and evaluation framework were derived through the synthesis of recurring concepts, components, and methodological patterns reported across the included studies. Particular emphasis was placed on identifying architectural components, behavioural intervention strategies, data processing mechanisms, and analytical methods relevant to adaptive health coaching and healthcare applications. Cross-domain studies were incorporated only where they contributed transferable behavioural intelligence, architectural designs, or technological capabilities applicable to IoB-enabled healthcare systems. To facilitate comparative analysis, the extracted evidence was organized into synthesized classification structures derived from recurring patterns observed across the reviewed literature. Directly extracted study attributes, including datasets, algorithms, evaluation metrics, architectural components, and technological characteristics, were mapped into these synthesized classifications. Synthesized elements are explicitly distinguished from directly extracted study findings throughout the Results and Discussion sections. This approach provides clear traceability between the underlying evidence base and the synthesized analytical structures presented in the review.

### 2.8 Quality assessment and risk-of-bias evaluation

To ensure the reliability and credibility of the synthesized evidence, a structured quality assessment and risk-of-bias evaluation was conducted for all studies included in the final review. The assessment was independently performed by two reviewers (PWCP and DPH), with any disagreements resolved through discussion and consensus. Where consensus could not be achieved, a third senior reviewer (MSS) was consulted to make the final assessment decision. The quality appraisal framework was developed based on methodological principles commonly adopted in systematic reviews of digital health, healthcare informatics, and AI-enabled healthcare systems, and was adapted to reflect the specific characteristics of IoB-enabled adaptive health coaching research. Accordingly, each included study was assessed across five key methodological domains: (i) Methodological Transparency, (ii) Dataset Quality and Reliability, (iii) Evaluation Rigor, (iv) Reproducibility, and (v) Clinical or Real-World Validation. These domains were selected because they capture the methodological, technical, and translational characteristics most frequently reported in IoB-enabled digital health research. For each domain, studies were evaluated using a qualitative three-level assessment scheme consisting of Yes, Partial, and No classifications. These ratings were assigned based on the extent to which each assessment criterion was addressed and reported within the study. The assessment was conducted to provide a structured evaluation of methodological quality rather than to generate a quantitative quality score. Consequently, the findings were synthesized qualitatively to identify recurring strengths, limitations, and potential sources of bias across the reviewed literature.

- Yes: The criterion was adequately addressed and clearly reported.
- Partial: The criterion was partially addressed or incompletely reported.
- No: The criterion was not addressed or insufficiently reported.

Particular attention was given to potential sources of bias, including incomplete methodological reporting, dataset selection bias, insufficient dataset documentation, limited validation procedures, lack of reproducibility information, and absence of real-world or clinical validation. For each included study, the five assessment domains were evaluated independently by the reviewers, and the resulting qualitative ratings were used to identify recurring methodological patterns and areas of potential bias across the evidence base. The explicit study-level ratings compiled across these domains directly influenced how evidence was weighted, synthesized, and interpreted throughout this review. Rather than treating all findings equally, claims regarding AI coaching efficacy, system performance, and IoB architectures from studies exhibiting a higher risk of bias or limited evaluation rigor (e.g., scoring “No” or “Partial” in Dataset Quality or Evaluation Rigor) were interpreted with heightened caution and framed primarily as preliminary, prototype, or conceptual frameworks. Conversely, digital health ecosystems backed by robust datasets and verified clinical validation (“Yes” ratings in both domains) served as the primary empirical baseline for synthesizing our final thematic taxonomy, ensuring that the review's structural conclusions prioritize methodologically sound and clinically reliable evidence.

Table 2 presents the quality assessment framework used to evaluate the methodological rigor of the included studies, while Table 3 summarizes the key aggregate observations and recurring methodological patterns identified through the quality appraisal process. The complete, itemized study-level quality assessment and risk-of-bias judgments for all 75 included studies are provided in Supplementary Table S3 (provided as Supplementary Material 4).

**Table 2:** Quality assessment framework applied to included studies.

Quality Domain	Assessment Question	No	Partial	Yes
Methodological Transparency	Is the study objective, system architecture, methodology, and workflow clearly described?	Not reported	Partially described	Clearly described
Dataset Quality and Reliability	Are the dataset source, characteristics, size, and representativeness adequately reported?	Insufficient information	Partially reported	Adequately reported
Evaluation Rigor	Are appropriate validation procedures, evaluation metrics, and comparative analyses reported?	Weak or absent evaluation	Limited evaluation	Comprehensive validation and evaluation procedures
Reproducibility	Are sufficient implementation details, experimental settings, and methodological procedures provided?	Insufficient information	Partially reproducible	Sufficiently reproducible
Clinical or Real-World Validation	Is there evidence of pilot deployment, user evaluation, clinical testing, or longitudinal validation?	No validation	Limited validation	Strong real-world or clinical validation

**Table 3:** Summary of quality assessment findings across included studies.

Assessment Domain	Synthesized Empirical Observation (Based on *n* = 75 mapped records)
Methodological Transparency	Methodological transparency was the strongest domain across the literature pool, with 95.9% (*n* = 71/75) of the studies adequately describing system architectures, data acquisition processes, and analytical workflows. Only 4.1% (*n* = 3/75) provided partial descriptions, facilitating strong interpretation and replication across the board.
Dataset Quality and Reliability	Dataset reporting was highly mixed; while 47.3% (*n* = 35/75) adequately reported their dataset sources and characteristics, a substantial 45.9% (*n* = 34/75) provided only partial details, frequently omitting demographic diversity, representativeness, or data collection protocols. A minor fraction 6.8%, (*n* = 5/75) contained insufficient information entirely.
Evaluation Rigor	Standard machine learning and system performance metrics were widely prioritized, with 71.6% (*n* = 53/75) demonstrating robust validation and evaluation procedures. However, 24.3% (*n* = 18/75) featured limited comparative evaluation or lacked external benchmarking, and 4.1% (*n* = 3/75) lacked baseline validation entirely.
Reproducibility	Reproducibility remains a systemic challenge in this field: only 39.2% (*n* = 29/75) provided sufficient implementation details and software configurations for exact replication. provide clear details. The majority 51.4%, (*n* = 38/75) were deemed only partially reproducible due to missing code or hyperparameter listings, and 9.5% (*n* = 7/75) lacked reproducibility information entirely.
Clinical or Real-World Validation	This domain exhibited the starkest implementation gap, with exactly half of the evidence base 50.0%, (*n* = 37/75) providing no real-world or clinical validation. Only 20.3% (*n* = 15/75) demonstrated strong real-world clinical testing or longitudinal pilot deployments, while 29.7% (*n* = 22/75) fell into the limited or conceptual validation category.

Overall, methodological transparency and evaluation rigor were the strongest domains across the included studies, whereas reproducibility and clinical validation exhibited the greatest limitations. These findings indicate that, despite substantial technological advancement, important challenges remain regarding reporting quality, implementation transparency, and real-world validation. The quality assessment findings were not used as exclusion criteria. Instead, they were considered during evidence synthesis to contextualize the strength, reliability, and limitations of the reported findings and to identify recurring methodological gaps across the literature. This review was conducted in accordance with the PRISMA 2020 guidelines. The completed PRISMA 2020 Checklist is provided as Supplementary Material 1.

## 3 Sensor technologies and data collection

Adaptive health coaching systems built on the Internet of Behaviours (IoB) rely heavily on the integration of sensor technologies and robust data collection pipelines. These systems must capture physiological, behavioural, and environmental data continuously to enable timely interventions. The evolution of wearables, healthcare IoT devices, smart home healthcare technologies, and medical sensors has facilitated the collection of rich, multi-modal data, providing the foundation for personalized behavioural analytics and real-time coaching (3–5). In the context of adaptive health coaching, sensors are the primary enablers of the perception layer, translating human actions and physical signals into digital information that feeds machine learning models for decision-making.

### 3.1 Wearable IMU sensors and smart devices

Wearable devices have become the cornerstone of IoB-enabled health monitoring. These devices often incorporate Inertial Measurement Units (IMUs) that combine accelerometers, gyroscopes, and magnetometers to detect motion patterns, posture, and activity intensity (32). For example, smartwatches and fitness trackers can continuously record step counts, heart rate variability, sleep patterns, and energy expenditure, which are essential inputs for behaviour-based coaching systems (7, 37). In clinical scenarios, continuous glucose monitors, ECG patches, and smart inhalers extend the scope of monitoring to chronic disease management, providing granular data that can trigger adaptive interventions for conditions like diabetes or COPD (24, 25).

**Figure 3:**
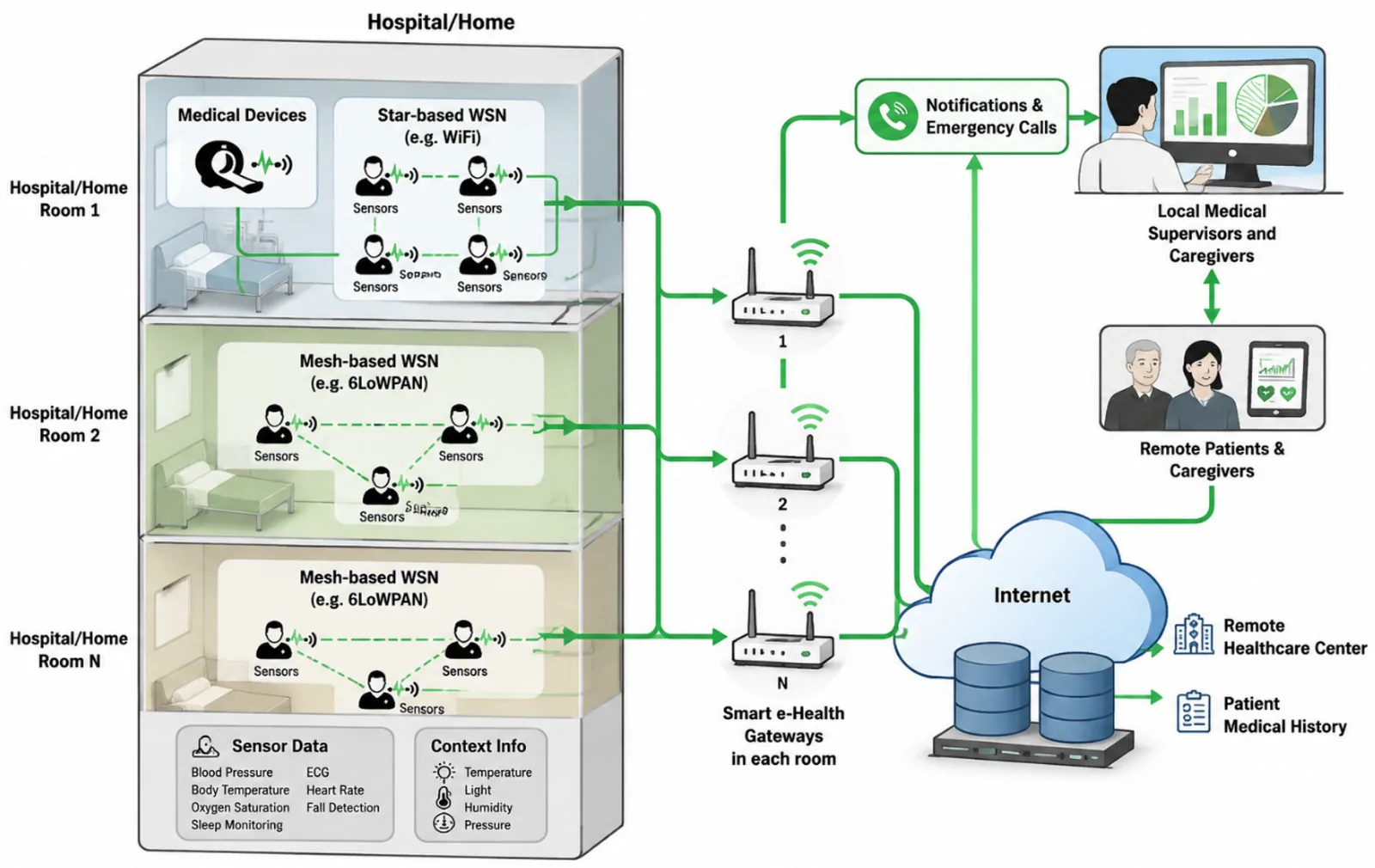
Components of IoT-based health monitoring system.

In Figure 3, the IoT-based health monitoring architecture illustrates how wearable sensors, medical devices, and wireless sensor networks populate the perception layer, continuously collecting physiological and contextual information. These data are transmitted through star-based and mesh-based communication networks to smart e-health gateways, where information is aggregated before being forwarded to cloud and healthcare management platforms adapted from (38). This architecture enables continuous monitoring, real-time feedback, and timely health interventions through communication between patients, caregivers, and healthcare providers. The sensing devices therefore function not only as data collection components but also as key enablers of adaptive behaviour-aware health coaching systems.

### 3.2 Physiological monitoring systems

Physiological monitoring is central to adaptive health coaching, as it enables the detection of critical deviations in vital signs. Devices such as smart ECG patches, pulse oximeters, blood pressure monitors, and glucose sensors generate continuous streams of health-related data that can be analysed for anomaly detection and risk prediction (26, 39). Integration of behavioural data with physiological measurements provides a more holistic understanding of health, enabling the system to distinguish between benign lifestyle fluctuations and potentially dangerous patterns. For instance, a spike in heart rate may be interpreted differently depending on whether it coincides with planned physical activity or unprovoked stress indicators (21, 27).

Moreover, advanced IoB-based platforms leverage multi-sensor fusion techniques to reduce false alarms and enhance the accuracy of health predictions. Data from physiological monitoring devices often undergo pre-processing at the edge, where noise filtering, anomaly detection, and signal normalization take place before integration into cloud-based health coaching models (28, 30). This edge processing is particularly important for latency-sensitive applications, such as fall detection or hypoglycaemia alerts, where a delay in decision-making could compromise patient safety.

### 3.3 Environmental and ambient sensing

While wearable and physiological sensors capture individual-centric data, environmental and ambient sensing provides contextual insights critical to behavioural interpretation. Sensors embedded in smart homes for assisted living, hospital rooms, rehabilitation facilities, and community healthcare settings monitor factors like room occupancy, temperature, humidity, and air quality, as well as motion and object interaction patterns (14, 33). In elderly care scenarios, ambient sensors can detect wandering behaviours, prolonged inactivity, or non-responsive states, triggering proactive interventions before a health event escalates (7, 20).

Environmental sensing also enhances behaviour prediction models by linking physiological responses to contextual triggers. For example, increased heart rate and respiratory rate might be attributed to heat exposure if the ambient temperature is high, rather than to stress or physical exertion (13). This multi-layered data context is what distinguishes IoB-based health coaching from conventional IoT health monitoring, as it captures the “why” behind behaviours rather than only the “what.”

### 3.4 Data collection methodologies and integration

Data collection in IoB-based adaptive health coaching systems involves seamless integration of multi-source data streams. Sensors and devices typically transmit data to edge gateways via Bluetooth Low Energy (BLE), Zigbee, or Wi-Fi, where initial pre-processing and anonymization may occur (28). Edge intelligence is increasingly employed to perform preliminary machine learning inference locally, reducing bandwidth usage and preserving privacy by keeping sensitive raw data on-device (2, 10). Once the data is sanitized and compressed, it is forwarded to cloud platforms for longitudinal storage, predictive analytics, and visualization for caregivers or healthcare providers.

**Figure 4:**
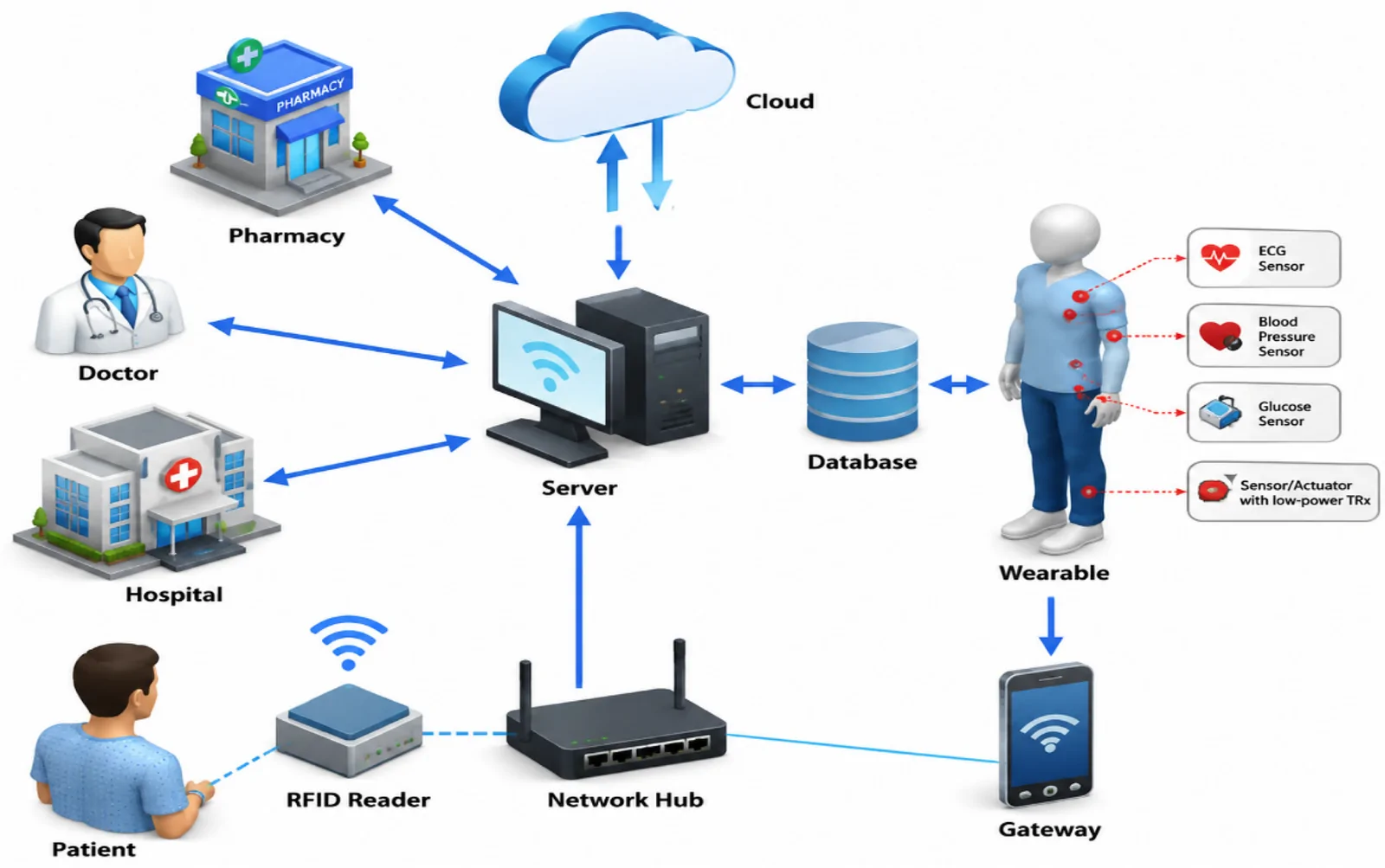
Application and challenges of IoT healthcare system.

Integration of data from multiple sources requires temporal alignment, feature fusion, and noise handling to ensure consistency and reliability. Advanced federated learning frameworks have been proposed to allow distributed model training across devices without centralizing raw data, addressing privacy concerns in sensitive health applications (9, 12). As illustrated in Figure 4, the IoT healthcare architecture follows a hierarchical data flow from wearable sensors and monitoring devices to gateway systems, databases, and server platforms, which subsequently communicate with cloud services and healthcare stakeholders, including hospitals, clinicians, and pharmacies adapted from (40). This integrated infrastructure supports continuous health monitoring, centralized data management, and the delivery of timely healthcare interventions. In practice, successful IoB deployments rely on robust interoperability frameworks that align with standards like HL7 FHIR for health data exchange, ensuring that data collected from diverse devices and vendors can be integrated seamlessly (8, 31). The integration of behavioural, physiological, and environmental data not only enables real-time monitoring but also supports predictive modelling, forming the foundation of adaptive health coaching.

Although IoB technologies have been investigated across multiple domains, this review focuses specifically on sensing and data collection mechanisms that directly support adaptive health coaching, chronic disease management, elderly care, and healthcare decision support. Non-healthcare sensing studies were considered only when their sensing architectures, data integration strategies, or behavioural analytics approaches demonstrated direct applicability to healthcare-oriented IoB systems.

## 4 Literature review

The reviewed literature positions Internet of Behaviours (IoB)-enabled adaptive health coaching at the intersection of behavioural science, connected sensing, artificial intelligence (AI), and digitally mediated intervention. Rather than treating these elements as independent technological streams, the evidence indicates an increasingly integrated ecosystem in which behavioural and physiological signals are captured continuously, interpreted through analytical models, and translated into context-sensitive feedback, alerts, recommendations, or coaching actions. Across the literature, however, the maturity of this integration is uneven. Some studies evaluate wearable monitoring, chronic disease management, and remote patient monitoring in empirical or real-world settings, whereas others propose conceptual architectures, prototype systems, simulations, or early-stage AI frameworks. Accordingly, this section synthesizes the literature thematically and comparatively, with emphasis on how sensing modalities, analytical methods, intervention mechanisms, populations, validation settings, and implementation constraints interact across the evidence base.

The synthesis is organized around conceptual and behavioural foundations; sensing and multimodal behavioural representation; AI-driven behavioural intelligence and adaptive intervention; healthcare applications and population-specific evidence; system architecture and interoperability; human AI coaching, adoption, ethics, and governance; and emerging directions and critical research gaps. This organization moves beyond a catalogue of technologies toward a critical account of where evidence is comparatively mature, where findings remain heterogeneous, and where claims are supported mainly by conceptual or prototype-level work. Detailed study-level characteristics remain in the supplementary evidence table, while the present section emphasizes recurring patterns, contrasts, and translational implications. Comparative interpretations of evidence maturity were derived from the study designs, evaluation settings, and validation characteristics reported in Supplementary Table S2 and were interpreted alongside the study-level quality assessment.

### 4.1 Conceptual foundations and behavioural frameworks

The IoB paradigm has evolved from the broader Internet of Things (IoT) by shifting analytical attention from connected objects alone to behavioural patterns generated through interactions among individuals, devices, environments, and digital services. IoB combines sensor-enabled observation, advanced analytics, and behavioural interpretation to infer, anticipate, and in some cases influence human actions (1). Early IoB applications were frequently discussed in marketing, education, and consumer analytics; healthcare applications increasingly focus on continuous behavioural sensing, personalized risk interpretation, and adaptive intervention. Wearable, ambient, physiological, and interaction data can be incorporated into feedback loops in which detected changes in activity, adherence, stress, sleep, medication use, or physiological status trigger personalized coaching or escalation pathways (3). This evolution is important because IoB-enabled health coaching differs conceptually from conventional remote monitoring. Traditional monitoring systems often terminate at data visualization or threshold-based alerts, whereas adaptive coaching seeks to connect observation with individualized behavioural action. The distinction is nevertheless blurred in the reviewed literature: some studies use the language of IoB while evaluating primarily sensing or prediction, whereas others implement a more complete closed loop linking data acquisition, behavioural inference, personalization, intervention delivery, and subsequent response monitoring. This heterogeneity suggests that IoB is better understood as a continuum of behavioural intelligence than as a single standardized architecture. Behaviour-change theory provides a second conceptual foundation. Adaptive interventions frequently draw on self-determination theory, motivational interviewing, goal-setting theory, the Transtheoretical Model, Social Cognitive Theory, and related behaviour-change taxonomies (23). These theories differ in emphasis: self-determination theory foregrounds autonomy and internalized motivation; motivational interviewing emphasizes collaborative resolution of ambivalence; goal-setting approaches structure progress through specific and responsive targets; and stage-based models adapt support to readiness for change. IoB systems can operationalize such principles by varying message timing, feedback intensity, goal difficulty, or intervention modality according to observed behaviour and inferred receptivity. Feedback following a lapse may shift from informational guidance to motivational prompting, while stable adherence may justify reduced prompting or reinforcement-oriented messages (41).

A recurring weakness, however, is the gap between behavioural theory and technical implementation. Many systems describe personalization through algorithmic adaptation but provide limited detail on how a named behavioural theory determines intervention logic. Conversely, theory-driven interventions may use relatively simple sensing or rule-based adaptation. Algorithmic personalization is not equivalent to theoretically grounded behaviour change: a high-performing prediction model may identify risk accurately without demonstrating that the resulting intervention improves adherence, self-efficacy, or sustained clinical outcomes. The strongest conceptual model is therefore a closed behavioural loop linking observation, interpretation, theory-informed intervention, response measurement, and iterative adaptation.

Table 4 condenses recurring component-level patterns into an integrated functional model. The categories represent thematic synthesis of architectures and system functions, while the representative components reflect technologies and mechanisms reported across the reviewed literature. The table highlights a central imbalance: sensing and predictive analytics are comparatively well developed, whereas theoretically explicit closed-loop adaptation and longitudinal clinical validation remain less consistently demonstrated.

**Table 4:** Functional synthesis of IoB-enabled adaptive health coaching systems.

Functional layer	Primary role	Representative components	Critical interpretation
Perception and sensing	Capture behavioural, physiological, interaction, and contextual signals	Wearables, IMUs, ECG/PPG, glucose sensors, ambient sensors, smartphones, voice and interaction logs	Rich context, but calibration, missing-data, burden, and privacy challenges
Data integration and representation	Synchronize heterogeneous streams	Edge preprocessing, timestamp alignment, sensor fusion, digital biomarkers, cloud synchronization	Multimodal integration improves context but weak standardization limits comparability
Behavioural intelligence	Infer states, patterns, risks, and deviations	ML, DL, temporal models, anomaly detection, multimodal fusion	Prediction is comparatively mature; external validation and interpretability remain uneven
Adaptive intervention	Translate inference into personalized action	JITAI, nudges, alerts, goal adaptation, conversational agents	Closed-loop intervention is less consistently validated than monitoring and prediction
Behaviour-change mechanisms	Support motivation, adherence, and sustained change	Goal setting, self-monitoring, motivational interviewing, gamification	Theory is often cited but mapping to algorithmic logic is inconsistent
Healthcare integration	Connect behavioural intelligence to care pathways	EHR, HL7/FHIR, dashboards, escalation workflows	Interoperability helps translation but workflow integration remains difficult
Trust, privacy, governance	Protect autonomy, fairness, and accountable use	Federated learning, encryption, consent, blockchain, XAI	Promising approaches; real-world deployment evidence remains limited
Evaluation	Assess technical, behavioural, clinical, and implementation performance	Accuracy, F1, AUC, latency, adherence, engagement, usability, clinical outcomes	Technical metrics dominate; longitudinal behavioural and clinical outcomes are less common

### 4.2 Sensing, data acquisition, and multimodal behavioural representation

Sensing constitutes the empirical foundation of IoB-enabled healthcare because behavioural adaptation depends on the availability, continuity, and contextual relevance of observed data. Three broad sensing families recur: wearable and mobile sensing, physiological monitoring, and environmental or ambient sensing. Wearable IMUs, smartwatches, and smartphones capture acceleration, posture, gait, mobility, and activity patterns, supporting fall detection, rehabilitation monitoring, and physical-activity coaching (7, 23). Physiological systems capture ECG, heart rate variability, blood pressure, glucose, and related biomarkers, providing more direct indicators of health state and risk (25, 39). Ambient sensors contribute contextual information through occupancy, movement, proximity, and environmental conditions, particularly in smart-home and elderly-care environments (8). These modalities are complementary rather than interchangeable. Wearables provide high-frequency person-centred information but may suffer from non-wear, battery limitations, motion artefacts, and adherence problems. Physiological sensing can increase clinical relevance but often requires stronger calibration, domain-specific interpretation, and attention to measurement reliability. Ambient sensing reduces user burden and can capture context continuously, yet may infer behaviour indirectly and raise concerns about surveillance and household privacy. Multimodal systems can combine movement, physiological state, and environmental context, but introduce synchronization errors, heterogeneous sampling rates, missingness, and more complex validation requirements. Data acquisition pipelines further differentiate system maturity. Many architectures combine real-time streaming with edge preprocessing and cloud-based storage or analytics. Wearables may transmit data through Bluetooth or lightweight messaging protocols to local gateways, where filtering, feature extraction, compression, or initial inference occurs before selected information is sent to cloud services. Federated learning offers an alternative in which sensitive data remain distributed while model updates are aggregated, reducing direct centralization of raw health information (2). Yet privacy preservation should not be inferred automatically from decentralization: model leakage, device heterogeneity, communication overhead, and uneven local data distributions remain relevant.

The reviewed datasets are similarly heterogeneous. Studies use wearable and physiological streams, public repositories, institution-specific clinical datasets, behavioural interaction logs, remote patient monitoring data, and synthetic or simulated environments. This diversity broadens application scope but weakens direct cross-study comparability. Defined patient cohorts and real-world longitudinal streams provide stronger contextual relevance, whereas synthetic data and controlled datasets are useful for feasibility testing but offer weaker evidence of external validity. Single-site or narrowly characterized datasets may produce high internal performance while leaving demographic representativeness and cross-population transfer uncertain. Dataset scale alone should therefore not be treated as a proxy for evidence quality; provenance, population coverage, missing-data handling, temporal structure, and external validation are equally important. Multimodal behavioural representation is a central analytical challenge. Activity recognition may rely on temporal segmentation of gait, sitting, sleeping, eating, or exercise patterns, while longitudinal models identify deviations from individual baselines (1, 42). Anomaly detection can flag prolonged inactivity, unexpected physiological changes, or deviations in medication-related routines (39). Combining IMU signals, heart rate, and room context may improve inference compared with a single modality (8), but performance gains must be weighed against additional complexity and the risk that missing one stream degrades the full model. The evidence supports multimodal sensing as a promising route to contextual intelligence while showing that robust deployment requires explicit treatment of sensor reliability, missingness, alignment, and generalizability.

### 4.3 AI-driven behavioural intelligence and adaptive intervention

AI and machine learning constitute the principal processing layer through which raw observations are transformed into behavioural states, risk estimates, forecasts, and intervention decisions. Conventional supervised methods including logistic regression, support vector machines, decision trees, and random forests remain relevant because they can perform effectively on structured clinical or behavioural features and may offer lower computational requirements and greater interpretability than deep architectures (39). Their limitations become more apparent when data are high-dimensional, multimodal, or strongly temporal. Deep learning addresses some of these limitations by learning hierarchical or sequential representations. Convolutional neural networks can extract complex patterns from ECG images and related signals, while recurrent networks and LSTM-based models capture temporal dependencies in activity, sleep, stress, and physiological trajectories (26). The comparative advantage of deep models is strongest where temporal or representational complexity exceeds what handcrafted features capture efficiently. However, deep models can require larger datasets, more computation, and more extensive tuning, while limited explainability can constrain clinical confidence. Ensemble and multimodal approaches seek to improve robustness by combining models or feature sources. Moustati and Gherabi (18, 43) illustrate the broader relevance of ensemble prediction across behavioural domains, with direct implications for systems integrating wearable, physiological, and ambient signals. Yet multimodal fusion should not be interpreted as inherently superior. Gains depend on data quality, alignment, complementarity, and validation design. In small or homogeneous datasets, a complex fusion model may increase overfitting without improving transportability. This is particularly important in IoB research, where behaviour can vary by age, culture, disease status, environment, and technology familiarity.

Behavioural intelligence also encompasses anomaly detection and temporal modelling. Rule-based thresholds remain useful for clinically interpretable events, whereas learned models can identify deviations that are difficult to specify *a priori*. Temporal forecasting can reveal progressive changes in sleep, mobility, or physiological status (42). The evidence suggests that monitoring and prediction are generally more mature than fully adaptive intervention. A model may detect inactivity or risk with high accuracy, but a closed-loop system must additionally determine whether to intervene, when to intervene, through which modality, with what intensity, and how to adapt after observing the user response. This distinction is central to just-in-time adaptive interventions and context-aware coaching. Adaptive systems may trigger activity nudges after prolonged inactivity, medication reminders following missed events, or tailored recommendations based on physiological response. Intervention timing can be adjusted according to inferred receptivity, while coaching tone may become more supportive, directive, or minimal depending on progress. Nevertheless, many studies evaluate only the technical component of this chain. Evidence of accurate prediction is stronger than evidence that algorithmically timed interventions produce sustained behavioural or clinical improvement. Claims about adaptive coaching should therefore distinguish technical feasibility from demonstrated intervention effectiveness. Real-time processing adds a further design constraint. Fall detection, arrhythmia alerts, and other time-sensitive applications require low-latency inference, making edge computing attractive. Lightweight models can execute locally, reduce bandwidth use, and limit transmission of raw sensitive data, while cloud infrastructure supports heavier training, longitudinal analytics, and cross-user aggregation (2). This edge–cloud division shapes which interventions can be delivered safely and promptly. The strongest evidence for edge intelligence is associated with applications in which response time is clinically or operationally meaningful; less urgent behavioural coaching may tolerate longer processing windows in exchange for richer contextual analysis.

Table 5 emphasizes that technology maturity is not uniform. Conventional prediction and sensor-based monitoring have broader empirical foundations, whereas explainable architectures, advanced adaptive intervention engines, and LLM-enabled coaching remain more dependent on emerging or early-stage evidence. The maturity descriptors are qualitative synthesis categories rather than a formal evidence-grading scale; they summarize the balance of validation settings and implementation depth observed across the included studies.

**Table 5:** Comparative synthesis of AI and IoB technology approaches.

Technology approach	Representative methods	Primary healthcare role	Key strength	Key limitation	Maturity
Conventional predictive ML	Logistic regression, SVM, RF, boosting	Risk stratification; adherence and behaviour prediction	Interpretability; lower compute	Feature dependence; limited complex temporal modelling	Moderate; application-dependent
Deep temporal learning	CNN, RNN, LSTM, autoencoders	Signal analysis; activity and longitudinal recognition	Complex nonlinear and temporal modelling	Data demand; opacity; overfitting risk	Mixed
Multimodal and ensemble intelligence	Sensor fusion, voting, stacked models	Integrated behavioural and physiological inference	Contextual richness; robustness potential	Synchronization and reproducibility challenges	Mixed
Edge and federated intelligence	Lightweight inference, FL, edge–cloud learning	Low-latency monitoring; privacy-sensitive analytics	Responsiveness; reduced raw-data centralization	Device heterogeneity; communication complexity	Emerging–moderate
Explainable and privacy-preserving AI	XAI, blockchain-linked governance, interpretable models	Trust, auditability, accountable support	Transparency and governance potential	Deployment complexity; limited real-world evidence	Emerging
Adaptive intervention intelligence	JITAI, feedback engines, DRL, personalization	Coaching, goal adaptation, behaviour-triggered intervention	Closed-loop personalization	Limited longitudinal and clinical validation	Emerging–mixed
Conversational and LLM-enabled coaching	Conversational agents, LLMs, motivational dialogue	Education, self-management, continuous coaching	Natural interaction; scalable personalization	Safety, hallucination, privacy, validation concerns	Early-stage

### 4.4 Healthcare applications and population-specific evidence

The healthcare applications of IoB span medication adherence, physical activity, chronic disease management, mental health, elderly care, remote monitoring, and clinical decision support. Across these domains, the role of IoB varies substantially. Some systems primarily observe behaviour; others predict risk; and a smaller subset closes the loop through adaptive coaching or intervention. A critical comparison therefore requires attention not only to application area but also to population, intervention type, validation setting, and outcome measure. Medication-adherence applications typically combine event logging, smart pillboxes, reminders, or behavioural monitoring to identify missed doses and prompt action (44). Their principal advantage is the direct connection between an observable behaviour and a defined intervention. However, medication-taking events can be difficult to verify reliably, and reminder delivery does not necessarily establish sustained adherence. Predictive models based on broader risk features may support individualized recommendations (39), but evidence of downstream clinical benefit requires more than classification performance. Physical-activity coaching commonly uses wearable data, adaptive goals, progress visualization, gamification, and feedback loops (23). Compared with medication adherence, these interventions often involve continuous behavioural targets rather than binary events. Wearables can quantify steps, duration, and intensity, allowing goals to be recalibrated as behaviour changes. Evidence is promising when sensing is combined with goal setting and motivational strategies, but long-term engagement remains a recurrent limitation. Improvements observed during short interventions may not persist after novelty or external reinforcement declines.

Chronic disease management represents one of the more clinically relevant application areas because sustained self-management is central to conditions such as diabetes, hypertension, COPD, and related long-term disorders. Sharma et al. (25) describe IoT-driven nutritional feedback that combines dietary information and glucose-related monitoring to support personalized recommendations. Across this domain, IoB can link physiological change with behaviour, enabling interventions that respond to both what a person does and how the body responds. Compared with general wellness applications, chronic disease studies may involve more defined clinical outcomes and patient populations; nevertheless, external validation, long-term follow-up, and integration with clinician workflows remain uneven. Mental health applications use combinations of physiological signals, self-report, interaction metadata, sentiment analysis, and conversational interfaces. Wearable stress indicators and heart-rate variability may contribute to state detection, while digital interfaces deliver breathing exercises, relaxation prompts, or escalation advice (45). Bobhate and Bhaladhare (46) explored a deep learning-based IoB framework for depression detection, illustrating the potential of behavioural data for early identification. Yet mental states are difficult to infer from indirect behavioural proxies, labels may be noisy, and false reassurance or inappropriate escalation can carry substantial consequences. Technical classification results should therefore not be equated with validated mental health diagnosis or therapeutic effectiveness.

Elderly care is one of the broadest IoB application areas, integrating wearables, ambient sensing, fall detection, assistive robotics, medication support, and caregiver communication. Ji et al. (7) discuss nursing robots and intelligent support, while Ioannidou et al. (8) highlight ontology-supported communication and interoperability. Studies of living conditions, medication risk, and polypharmacy further show how behavioural and contextual information may inform support for older adults (39, 47, 48). Relative to some emerging domains, elderly care benefits from multiple empirical and applied settings. However, older populations are heterogeneous in frailty, cognition, digital literacy, social support, and comorbidity; findings from narrowly selected cohorts should not be generalized uncritically. Remote patient monitoring cuts across disease and population categories. Its comparatively mature use cases involve continuous physiological or behavioural observation, threshold-based alerts, and clinician dashboards. The transition from remote monitoring to IoB-enabled adaptive coaching occurs when observed patterns influence personalized recommendations or intervention timing. Remote data collection alone does not demonstrate behavioural intelligence. The literature suggests stronger empirical support for monitoring and early detection than for fully autonomous behaviour modification.

Table 6 demonstrates that application domains differ not only in technology but in the meaning of a successful outcome. Monitoring studies frequently prioritize accuracy, sensitivity, specificity, F1-score, latency, and alert performance, whereas intervention studies additionally assess adherence, engagement, usability, health literacy, self-management, or clinical change. This distinction is methodologically important: strong technical performance may support feasibility but does not by itself establish sustained behaviour change or improved health outcomes.

**Table 6:** Comparative synthesis of healthcare application domains.

Application domain	Population/context	Dominant IoB components	Intervention	Typical outcomes	Critical evidence pattern
Medication adherence	Long-term medication and chronic disease populations	Smart pillboxes, logs, reminders, risk prediction	Event-triggered reminders; tailored prompts	Adherence, missed doses, alert response	Moderate; verification and long-term persistence remain issues
Physical activity and wellness	General adults, sedentary users, rehabilitation and fitness populations	Wearables, IMUs, activity analytics	Adaptive goals, gamification, feedback	Steps, activity, engagement, goal attainment	Moderate; sustained engagement varies
Chronic disease management	Diabetes, hypertension, COPD and other long-term conditions	Physiological sensing, RPM, behavioural logs	Personalized self-management and risk feedback	Biomarkers, adherence, self-management, clinical outcomes	Comparatively stronger in selected applications
Mental health support	Users with stress, mood, or depression-related needs	HRV, self-report, interaction logs, conversational AI	Prompts, coping support, escalation	Classification, stress, engagement, symptom scales	Mixed; clinical interpretation requires caution
Elderly and assisted living	Community-dwelling older adults, care recipients	Ambient sensing, wearables, robots, ontologies	Fall alerts, assistance, coaching, caregiver escalation	Safety, mobility, usability, care outcomes	Moderate and application-dependent
Remote patient monitoring	Patients managed at home or across distributed care	Connected medical devices, edge/cloud analytics	Alerts, feedback, clinician escalation	Detection, latency, physiological and service outcomes	Comparatively mature for monitoring; adaptive coaching less established
Clinical decision support	Patients and clinicians in diagnostic or prognostic workflows	AI models, EHR-linked analytics, IoT data	Risk stratification and decision support	Accuracy, AUC, sensitivity, workflow outcomes	Mixed; external and prospective validation uneven

### 4.5 System architecture, edge cloud integration, and interoperability

IoB-enabled health coaching systems commonly adopt layered architectures comprising perception, communication, processing, intelligence, application, and governance functions. Earlier IoT health architectures established the role of sensors, gateways, remote services, and clinical access (38, 49). In contemporary IoB systems, these layers are extended by behavioural inference and adaptive intervention. Wearables and ambient devices capture physiological and contextual signals; gateways or edge nodes perform preprocessing and selected inference; cloud platforms support storage, model training, longitudinal analytics, and dashboards; and application layers deliver feedback to users, caregivers, or clinicians (3). The principal architectural trade-off concerns where computation occurs. Edge processing supports low-latency response and can reduce transmission of raw sensitive data. It is particularly relevant to fall detection, arrhythmia alerts, and other time-sensitive applications. Cloud computing offers greater computational capacity, centralized model management, and cross-user longitudinal analysis. Hybrid edge–cloud designs therefore dominate many proposed architectures, with local inference for urgent or privacy-sensitive functions and cloud services for heavier analytics and retraining (2). Practical maturity depends on network reliability, device capability, update management, cybersecurity, and validation under real operating conditions.

**Figure 5:**
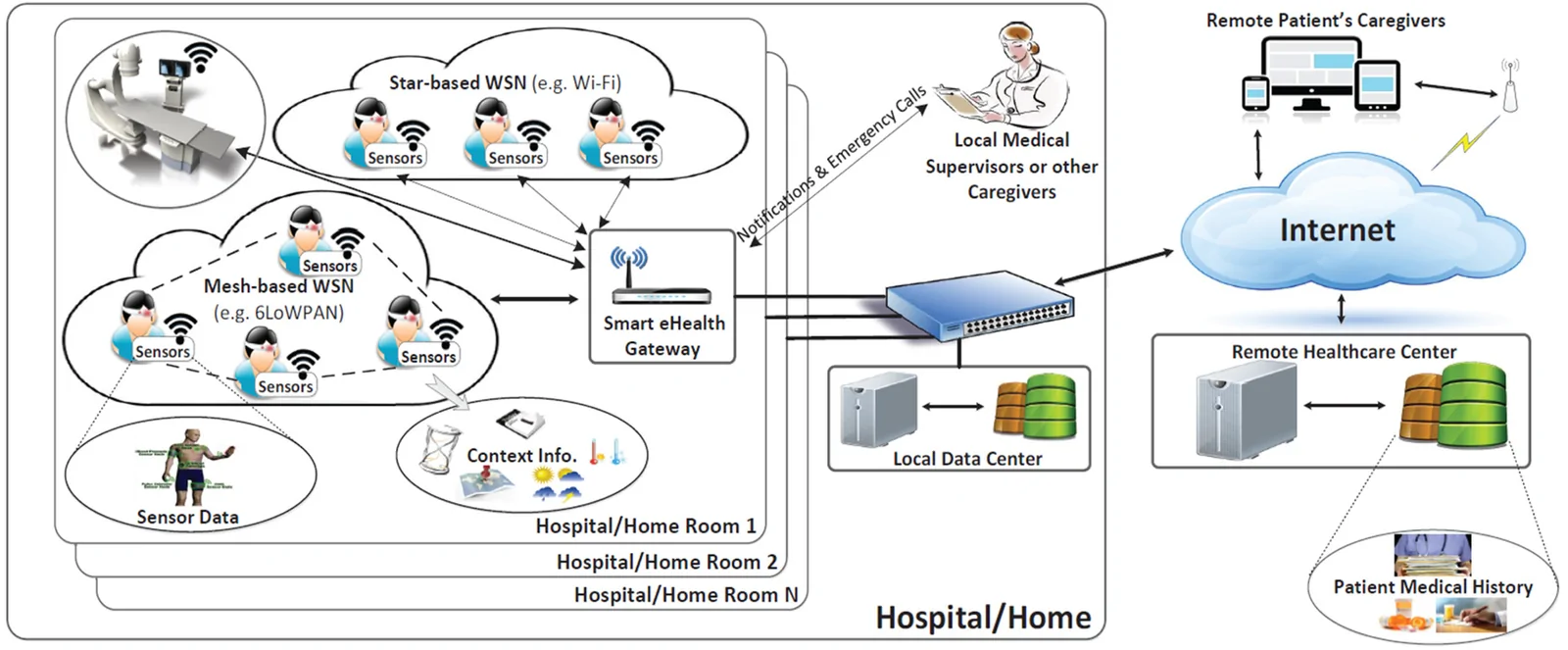
IoT-based health monitoring architecture diagram.

As shown in Figure 5, physiological, behavioural, and contextual data are collected through medical devices and wireless sensor networks operating in hospital and home environments. These data are transmitted through smart eHealth gateways to local or remote healthcare services and caregiver networks, supporting continuous monitoring, data aggregation, access to patient information, and timely intervention adapted from (49). This architecture provides a foundation for adaptive health coaching; however, the critical extension from conventional IoT monitoring to IoB lies in the behavioural intelligence layer, which interprets observed patterns and supports context-aware adaptive action rather than passive data transmission alone. Communication protocols and interoperability determine whether this architecture can operate across heterogeneous devices and clinical systems. Lightweight protocols such as MQTT can support resource-constrained messaging, while RESTful interfaces facilitate service integration. Healthcare interoperability standards such as HL7 and FHIR support exchange with electronic health records and clinical dashboards, and semantic ontologies such as HERON can improve consistency across devices and care contexts (8). Yet standards compliance does not guarantee meaningful workflow integration. Data may be technically interoperable while remaining poorly aligned with clinical responsibilities, escalation procedures, or decision-making practices.

**Figure 6:**
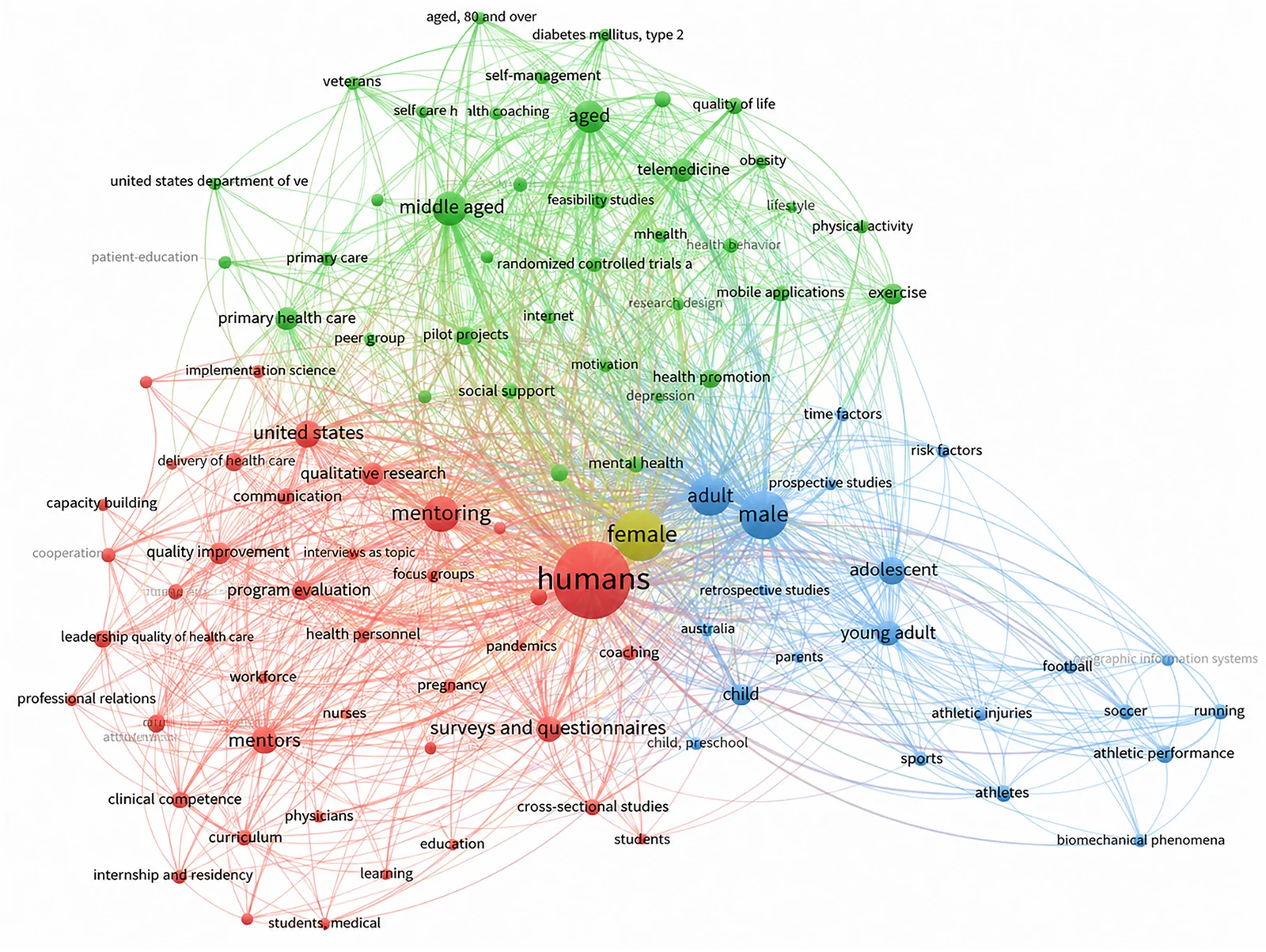
Network diagram.

Figure 6 illustrates the distributed connectivity among sensing devices, gateways, edge resources, cloud services, and end users. Such distribution creates resilience and scalability opportunities but also expands the attack surface and complicates identity management, access control, software updates, and accountability.

Cloud platforms support model training, data warehousing, dashboards, retrospective analysis, and personalization across longitudinal records (3). Their value is particularly evident when behavioural patterns must be interpreted over days or months rather than seconds. However, centralized aggregation intensifies concerns about data minimization, secondary use, consent, and cross-border governance. Federated and privacy-preserving approaches seek to reduce these risks, but their real-world performance must be evaluated under non-identically distributed user data, intermittent connectivity, and heterogeneous devices.

**Figure 7:**
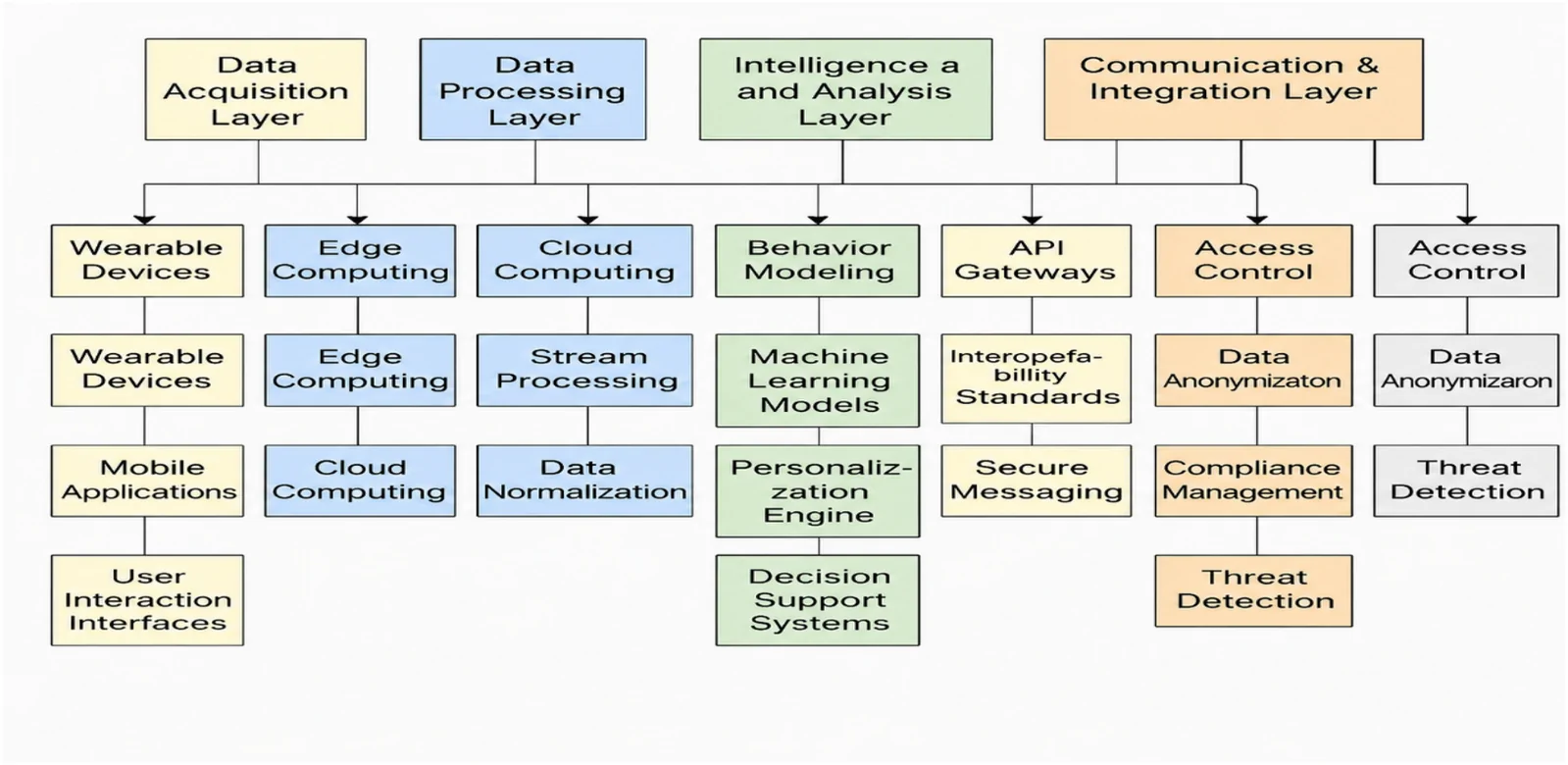
Proposed IoB based adaptive health coaching system architecture.

As illustrated in Figure 7, the proposed IoB-based adaptive health coaching architecture integrates wearable devices, edge–cloud processing, machine-learning-based behavioural analysis, personalization engines, decision-support mechanisms, and secure communication within a closed-loop framework. The principal challenge is not the availability of individual components, but their validated integration into a reliable, clinically meaningful, and ethically governed system. Evidence maturity also varies across architectural layers: sensing, connectivity, and cloud analytics are comparatively established, whereas seamless organizational interoperability, closed-loop adaptive intervention, and longitudinal validation of complete end-to-end systems remain less mature. Thus, prototype functionality should be interpreted as evidence of feasibility rather than evidence of safe or effective healthcare deployment.

### 4.6 Human AI coaching, adoption, ethics, and governance

The increasing use of AI in health coaching has intensified interest in hybrid human AI models. These systems combine automated pattern analysis and scalable routine interaction with human contextual judgment, empathy, and accountability. Earlier digital health work suggests that human oversight can strengthen trust and satisfaction, particularly where chronic disease or lifestyle change requires interpretation beyond a narrow sensor signal (41, 50). AI may generate reminders, summaries, risk flags, or routine coaching, while human coaches address ambiguity, complex motivation, or clinically sensitive decisions. Conversational intelligence extends this model by allowing users to interact through natural language. Jörke et al. (51) evaluated GPTCoach for physical-activity behaviour change, illustrating how conversational agents can combine motivational interviewing principles with wearable information. Ilukpitiya et al. (52) similarly contribute to the emerging discussion of adaptive, human-like interaction. The potential advantages are accessibility, continuity, and scalable personalization. Nevertheless, conversational fluency should not be confused with clinical reliability. LLM-enabled systems introduce risks related to hallucination, inconsistent advice, privacy, prompt sensitivity, and unclear accountability. Their evidence base should therefore be interpreted as promising but less mature than established monitoring applications. Wearables and digital therapeutics further blur the boundary between monitoring and intervention. Continuous measurement of activity, sleep, heart rate, and stress-related signals can support personalized content, while goal setting, gamification, and feedback loops may increase engagement (6). Digital therapeutics can incorporate real-time behavioural information to adapt modules for diabetes, stress, or other conditions. Yet regulatory status and evidence requirements vary substantially. Systems that influence clinical management require stronger transparency, validation, and risk controls than general wellness applications, and software-as-a-medical-device considerations reinforce the need to distinguish supportive coaching from clinical decision-making.

Privacy and ethics are not peripheral constraints but core determinants of IoB legitimacy. Behavioural data can reveal routines, vulnerabilities, location patterns, emotional states, and social interactions even when conventional medical records are absent. Oleribe et al. (53) emphasize ethical issues surrounding AI adoption in healthcare, including autonomy, fairness, transparency, and accountability. In IoB systems, informed consent is complicated by continuous sensing and secondary inference: users may consent to collection of activity data without anticipating that the same data could be used to infer stress, adherence, or risk. Privacy-preserving technologies provide partial responses. Federated learning can keep raw data local while sharing model updates; blockchain can support auditability and consent records; and explainable AI can make selected model outputs more interpretable. The literature includes federated and edge-oriented approaches, including work discussed by Chien et al. (2) and Sruthi Sai Prabha et al. (54). However, technological safeguards do not eliminate governance questions. Federated models can still leak information, blockchain immutability may conflict with data-erasure expectations, and *post hoc* explanations may not faithfully represent complex model reasoning. Ethical claims should therefore be grounded in demonstrated system properties rather than the mere presence of a privacy or XAI technique. Adoption is similarly multidimensional. User trust, perceived usefulness, digital literacy, technology type, generational differences, burden, accessibility, clinician attitudes, organizational readiness, and workflow compatibility influence whether a technically effective system is used consistently (55). Frameworks such as NASSS and the Theoretical Domains Framework can structure analysis of implementation complexity and behavioural determinants. Co-design, stakeholder engagement, iterative testing, and technology-use support may improve retention, but the evidence remains context-dependent. An intervention acceptable to younger technology-familiar users may not transfer directly to frail older adults or populations with limited connectivity. These findings support a human-centred interpretation of IoB maturity. The most impactful systems are unlikely to be those with the most complex models alone; rather, they are those that align sensing burden, predictive value, intervention timing, user preferences, clinical workflows, and governance. Human oversight remains particularly important where model uncertainty is high, consequences are clinically significant, or behavioural recommendations require contextual judgment. Hybrid coaching should therefore be viewed not as a temporary compromise before full automation, but as a potentially durable design strategy for accountable and relational healthcare.

### 4.7 Emerging directions and critical research gaps

Emerging IoB research is extending toward digital twins, immersive environments, ambient intelligence, advanced behavioural simulation, and conversational AI. Digital twins can model dynamic patient or system states and support simulation of interventions before real-world application (42, 56). Metaverse-related and immersive approaches similarly propose new environments for behavioural engagement and preventive care. These directions are conceptually important because they expand IoB from reactive monitoring toward predictive and simulated decision support. However, much of the evidence remains at conceptual, prototype, or simulation level, and claims of clinical effectiveness should therefore be framed cautiously. Conversational and multimodal wearable systems represent another emerging direction. Naccarelli et al. (57) describe technical architecture for virtual coaching in older adults, illustrating how sensing, dialogue, and distributed services can be combined. Voice-first and LLM-enabled devices may reduce interaction barriers for users who find complex mobile interfaces difficult. Yet the same systems raise unresolved questions about speech privacy, model reliability, personalization boundaries, and escalation when users express clinically significant symptoms. Their translational value will depend on prospective evaluation rather than technical novelty alone. A first major research gap is longitudinal and clinical validation. Across the evidence base, technical evaluations, controlled experiments, simulations, and prototypes remain common, whereas long-term clinical studies and real-world deployments are less consistently represented. This limits conclusions about sustained adherence, behavioural maintenance, adverse effects, and clinical effectiveness. Future studies should distinguish short-term engagement from durable behaviour change and report whether improvements persist after intensive prompting or novelty effects diminish. A second gap concerns population and dataset generalizability. Many systems are developed using narrowly characterized cohorts, private institutional data, public benchmark datasets, or simulated environments. Behaviour is highly context-dependent, and models trained in one age group, culture, healthcare system, or device ecosystem may not transfer reliably. Cross-population validation should therefore be treated as a core requirement rather than an optional extension. Reporting should include dataset provenance, demographic composition, missingness, sensor characteristics, recruitment context, and external validation. A third gap is the imbalance between technical and behavioural outcome measures. Accuracy, F1-score, AUC, sensitivity, latency, and resource efficiency are essential for system validation, but they do not demonstrate that an intervention changes behaviour or improves health. Future IoB coaching research should integrate technical endpoints with adherence, engagement, self-efficacy, quality of life, clinically meaningful biomarkers, service utilization, and adverse outcomes where appropriate. Mixed-method evaluation can further reveal why an intervention succeeds or fails. A fourth gap concerns reproducibility and implementation transparency. Complex IoB systems combine sensors, preprocessing, feature engineering, models, thresholds, intervention rules, and communication infrastructure. Incomplete reporting at any stage can prevent replication. The field would benefit from clearer descriptions of device specifications, sampling rates, preprocessing, missing-data strategies, hyperparameters, software versions, intervention logic, and deployment context. Where privacy permits, code, model cards, data dictionaries, and synthetic reproducibility resources should be made available. A fifth gap lies in theory-to-system traceability. Behavioural theories are frequently invoked, but the mechanism through which theory changes algorithmic decisions is often unclear. Future studies should specify how theoretical constructs map to measurable variables, how those variables influence intervention selection or timing, and how behavioural mediators are evaluated. Such traceability would help distinguish genuine theory-informed adaptation from generic personalization. A sixth gap concerns privacy, fairness, and governance under continuous behavioural inference. IoB systems may derive sensitive conclusions from apparently innocuous signals, creating risks that conventional consent models do not fully address. Future work should examine dynamic consent, inference transparency, fairness across demographic groups, model drift, cybersecurity, and governance of secondary data use. Privacy-preserving learning, explainability, and blockchain may contribute, but these techniques require empirical evaluation in realistic healthcare environments rather than purely architectural justification. Finally, implementation research must address interoperability and organizational readiness. Even accurate and acceptable systems may fail if they do not integrate with clinical workflows, reimbursement structures, professional responsibilities, and escalation pathways. Standards such as HL7/FHIR can facilitate technical exchange, but successful deployment also requires clarity regarding who receives alerts, who is responsible for action, how false alarms are managed, and how patient-generated data influence clinical decisions. Future evaluations should therefore incorporate implementation outcomes alongside technical and clinical performance.

### 4.8 Integrative synthesis and research implications

Taken together, the literature supports IoB-enabled adaptive health coaching as a convergent research area rather than a single mature intervention class. The strongest and most consistent evidence concerns the feasibility of continuous sensing, behavioural or physiological pattern recognition, remote monitoring, and selected predictive applications. Wearable and sensor-based systems can capture meaningful longitudinal signals, and AI models can identify patterns relevant to activity, risk, adherence, and physiological change. In elderly care, remote patient monitoring, and selected chronic disease applications, the evidence includes empirical evaluations and applied settings that provide comparatively stronger support than purely conceptual work. Evidence for personalized coaching and adaptive behavioural intervention is promising but more heterogeneous. Some systems connect real-time data to goals, prompts, recommendations, or conversational support, yet intervention designs, populations, and outcome measures vary substantially. Monitoring-oriented studies tend to report technical metrics, whereas intervention-oriented studies assess engagement, adherence, usability, behavioural response, or clinical outcomes. Because these endpoints are not interchangeable, high predictive accuracy should not be interpreted as proof of sustained behaviour change. The field currently provides stronger support for technical feasibility and behavioural inference than for long-term clinical effectiveness. Evidence is less mature for digital twins, advanced behavioural simulation, some explainable IoB architectures, and emerging LLM-enabled coaching systems. These areas offer substantial conceptual and technical promise, but current conclusions are more often based on simulations, prototypes, controlled experiments, or early-stage framework evaluations. They should therefore be described as emerging rather than established. This distinction is essential for avoiding overstatement and aligning claims with the maturity of the supporting evidence.

The comparative synthesis also reveals that technological sophistication does not resolve translational barriers automatically. More sensors can increase context but also burden, missingness, and privacy risk. More complex AI can improve representation but reduce interpretability and reproducibility. More frequent interventions can increase contact but also produce fatigue or disengagement. Cloud integration can enable scale but intensify governance concerns, while edge processing can improve latency but introduce device constraints. Progress in this field will therefore depend on evidence that optimizes these trade-offs rather than simply adding technical components. Future research should prioritize longitudinal and multi-site validation; representative and transparently documented datasets; explicit mapping between behavioural theory and adaptive logic; integrated technical, behavioural, and clinical outcomes; privacy and fairness evaluation under realistic deployment conditions; and interoperable human AI workflows. Hybrid coaching models are particularly important because they can combine scalable automated monitoring with human judgment where uncertainty, empathy, or clinical responsibility is central. Similarly, conversational agents and LLMs should be evaluated through rigorous safety and effectiveness studies before broad claims of therapeutic impact are made. IoB-enabled adaptive health coaching has progressed from isolated sensing and rule-based alerts toward multimodal behavioural intelligence, edge cloud analytics, personalized feedback, and conversational interaction. Nevertheless, the evidence base remains heterogeneous in design, population, dataset, intervention type, validation setting, and outcome measurement. The most defensible conclusion is therefore not that IoB has uniformly transformed healthcare, but that it has established a credible technical and empirical foundation in selected monitoring and chronic-care contexts while more advanced adaptive, immersive, and generative-AI applications remain at earlier stages of translational maturity.

## 5 Discussion

The discussion indicates that IoB-enabled adaptive health coaching is developing through the convergence of multimodal behavioural sensing, AI-based inference, connected healthcare infrastructure, and adaptive intervention mechanisms (1, 2, 7, 11, 39). However, the reviewed evidence remains heterogeneous across study designs, populations, datasets, intervention types, validation settings, and outcome measures. Accordingly, this discussion focuses on areas with comparatively stronger empirical support, domains where evidence remains preliminary, and methodological and translational barriers to broader healthcare implementation (18, 23, 58, 59). Non-healthcare studies are considered only where they contribute transferable behavioural intelligence, architectural mechanisms, or analytical capabilities directly relevant to IoB-enabled healthcare systems.

### 5.1 Prediction to adaptive health coaching

The core of IoB-enabled health systems lies in their ability to predict and personalize interventions. Machine learning techniques such as logistic regression, support vector machines, and ensemble methods allow systems to model patient-specific risks, identify anomalous behaviours, and generate adaptive feedback (19, 30, 39, 43, 59). For instance, continuous ECG monitoring analysed with deep learning can detect arrhythmias early, and glucose sensor data can be used for proactive dietary and medication adjustments (24, 26, 60). Personalization is enhanced by behavioural science frameworks. Systems grounded in Self-Determination Theory and Transtheoretical Models dynamically adjust coaching intensity based on a user's stage of behavioural readiness (1, 22, 23, 61). This approach mirrors the logic of Just-in-Time Adaptive Interventions (JITAI), where timely notifications improve adherence in medication management and lifestyle interventions (5, 14, 45). Moreover, predictive IoB systems shift from reactive alerts to anticipatory guidance by combining longitudinal behavioural data and contextual cues (14, 27, 62). This anticipatory capacity underpins modern chronic disease management, fall prevention, and mental health support systems (21, 63, 64).

### 5.2 Elderly care and ambient behavioural intelligence

Elderly care emerged as one of the comparatively more developed application domains, leveraging ambient intelligence, robotics, and multi-sensor fusion to promote safe and healthy aging (7, 8, 20, 47, 48). Beyond individual level monitoring, data driven approaches have also been applied to elderly care planning and service environments, highlighting the broader role of big-data analytics in supporting aging populations (65). Ambient sensors such as infrared motion detectors and environmental monitors detect inactivity or hazardous patterns, while wearable IMUs track gait and mobility (4, 32, 33). Nursing robots and assistive social robots can extend IoB-enabled elderly care through health assistance, reminders, and interactive support, while their successful adoption depends on factors such as perceived usefulness, usability, and user acceptance (21, 62, 66, 67). Frameworks like HERON ontology facilitate semantic integration among devices, enabling contextual decision-making across edge and cloud layers (8, 12, 68). Ethically, elderly care IoB demands privacy-preserving data practices. The integration of federated learning, blockchain, and XAI supports localized processing while maintaining trust (10, 11, 28, 69). This privacy-by-design model aligns with regulatory requirements and strengthens adoption among vulnerable populations (70–72).

### 5.3 Ethical design, privacy, and explainability

Ethics and privacy remain central challenges in IoB adoption. Systems that continuously collect physiological and behavioural data face scrutiny over data ownership, consent, and explainability (17, 34, 73). Blockchain-based ledgers and dynamic consent mechanisms provide auditability and user control over personal data (10, 69). Moreover, the integration of Explainable AI (XAI) frameworks ensures that clinicians and end-users can interpret system recommendations, which improves trust and aligns with regulatory and clinical validation processes (11, 74). The combination of privacy-preserving analytics and ethical frameworks is vital for scaling IoB in sensitive domains such as eldercare and mental health monitoring (14, 64, 75). However, much of the evidence supporting blockchain, XAI, and privacy-preserving architectures remained conceptual, prototype-based, or technically evaluated, with limited demonstration that these mechanisms improved trust, adoption, or governance outcomes in longitudinal healthcare settings.

### 5.4 Translational implications of architecture, performance, and scalability

IoB health systems typically follow a layered architecture: perception (sensors), connectivity (IoT protocols), processing (ML/AI models), and application (coaching or dashboards) (38, 40, 49, 68, 76). Edge computing reduces latency for time-sensitive tasks like fall detection, while cloud platforms aggregate longitudinal data for deep analysis and retraining (2, 24, 28). Interoperability standards such as HL7/FHIR and IoT ontologies ensure seamless integration with clinical workflows and smart home ecosystems (5, 8, 77). Performance evaluation involves technical, clinical, and behavioural metrics, including accuracy, precision, engagement rate, and health outcome improvement (30, 32). The proposed layered architecture emerged from recurring patterns observed across the reviewed studies. Although implementation details varied among individual systems, most incorporated sensing, communication, data processing, and intervention functionalities. These common elements were synthesized into a unified conceptual model to facilitate the understanding and comparison of IoB-enabled adaptive health coaching systems. The architecture should therefore be interpreted as a synthesized reference model derived from the reviewed literature rather than a universally established standard.

### 5.5 Critical synthesis of evidence strength and maturity

The included studies demonstrated substantial heterogeneity in study design and validation setting. Experimental and system-development studies evaluated IoB-enabled monitoring, predictive analytics, and adaptive interventions through laboratory experiments, prototype implementations, testbeds, and algorithmic evaluations, whereas a smaller group employed pilot deployments, field evaluations, randomized or quasi-experimental designs, and clinical or real-world settings. Studies in remote patient monitoring, chronic disease management, elderly care, and wearable health monitoring generally showed greater empirical maturity because several incorporated patient cohorts, real-world sensor data, community settings, or clinical evaluation. In contrast, emerging applications involving digital twins, advanced behavioural simulation, explainable IoT architectures, and some LLM- or AI-driven adaptive systems were more frequently evaluated through prototypes, simulations, or controlled experimental settings. The populations and datasets also varied considerably across the evidence base. Applied studies included older adults, patients with chronic conditions such as hypertension, diabetes, chronic kidney disease, and COPD, as well as users of remote monitoring and digital health systems. Other studies relied on public repositories, wearable and physiological sensor data, institution-specific datasets, behavioural logs, synthetic data, or simulated patient environments. Studies involving defined patient populations and real-world healthcare data provided stronger contextual relevance, whereas reliance on synthetic, simulated, single-site, or narrowly characterized datasets limited external validity and cross-population generalizability. The heterogeneity of dataset sources and reporting practices also constrained direct comparison and reproducibility across studies. Intervention types ranged from passive monitoring and anomaly detection to behavioural prediction, personalized recommendation, conversational coaching, adaptive feedback, and closed-loop health interventions. Monitoring-oriented and algorithmic studies predominantly reported technical outcomes, including accuracy, sensitivity, specificity, communication efficiency, latency, anomaly-detection performance, and predictive discrimination. In contrast, intervention-oriented studies more frequently assessed adherence, engagement, health literacy, self-management, usability, behavioural response, or physiological and clinical outcomes. This distinction is important because strong technical performance does not necessarily demonstrate sustained behaviour change or clinical effectiveness. Similarly, systems evaluated through prototype or simulation settings provide evidence of technical feasibility but weaker support for long-term healthcare impact. The comparative patterns summarized in Table 7 were derived from the study-level characteristics reported in Supplementary Table S2 and interpreted alongside the study-level quality and risk-of-bias assessments.

**Table 7:** Cross-study comparative synthesis and maturity across the included studies.

Comparative dimension	Main pattern across included studies	Evidence status	Critical interpretation
Study design	Experimental, system-development, algorithmic, prototype, observational, survey, pilot, and clinical designs	Mixed	Technical and developmental studies are more prominent than rigorous longitudinal clinical evaluations
Population	Older adults, chronic disease patients, remote-monitoring users, and technology-specific cohorts	Moderate	Applied populations provide healthcare relevance, but cross-population and demographic generalizability remain limited
Dataset	Wearable/physiological data, clinical cohorts, behavioural logs, public repositories, private datasets, and synthetic/simulated data	Mixed	Heterogeneous and narrowly characterized datasets constrain reproducibility and external validity
Intervention type	Monitoring, anomaly detection, behavioural prediction, personalized recommendation, coaching, and adaptive feedback	Mixed	Monitoring and prediction are more mature than fully closed-loop adaptive interventions
Validation setting	Laboratory/testbed, simulation, prototype, pilot, field, community, and clinical settings	Limited Moderate	Real-world and longitudinal clinical validation remain less common than technical evaluation
Application domain	Elderly care, remote monitoring, chronic disease management, mental health, wellness, and adaptive healthcare	Moderate and domain-dependent	Established monitoring domains show stronger empirical maturity than emerging AI-enabled applications
Outcome measures	Accuracy, sensitivity, specificity, F1-score, latency, efficiency, adherence, engagement, usability, and clinical outcomes	Mixed	Technical outcomes dominate; sustained behavioural and clinical outcomes are less consistently assessed

Overall, the evidence was comparatively stronger for continuous wearable and sensor-based monitoring, remote patient monitoring, elderly care, and chronic disease management, where the reviewed literature included empirical evaluations, pilot deployments, field studies, and clinical interventions. Evidence for personalized coaching and adaptive behavioural interventions was promising but more heterogeneous because validation ranged from randomized and quasi-experimental studies to limited prototype evaluations. By contrast, conclusions regarding digital twins, advanced behavioural simulation, explainable IoT frameworks, and some emerging AI- or LLM-enabled systems were supported predominantly by early-stage experimental, prototype, or simulation-based evidence. Accordingly, the reviewed literature provides stronger support for the technical feasibility of continuous monitoring and behavioural inference than for sustained behaviour change, long-term clinical effectiveness, or large-scale healthcare implementation.

## 6 Conclusions and recommendations

The synthesis of the reviewed evidence indicates that IoB-enabled adaptive health coaching is an evolving interdisciplinary field integrating wearable and ambient sensing, behavioural analytics, edge–cloud infrastructure, and artificial intelligence to support personalized and context-aware health interventions. Across the included studies, comparatively stronger empirical support was observed for continuous monitoring, behavioural and physiological pattern recognition, remote patient monitoring, elderly care, and selected chronic disease applications. In contrast, evidence for advanced adaptive coaching, digital twins, behavioural simulation, and emerging AI- or LLM-enabled systems remained more heterogeneous and was frequently derived from prototype, simulation-based, or early-stage evaluations. Recurring multi-layer architectures comprising sensing, communication, processing, intelligence, and intervention functions informed the synthesized reference architecture proposed in this review. However, broader translation remains constrained by limited longitudinal and clinical validation, interoperability challenges, privacy and governance concerns, reproducibility limitations, and inconsistent evidence of sustained user engagement and behavioural change (1, 2, 8, 11, 59). Accordingly, the following recommendations are framed around the key evidence gaps and translational priorities identified through the review.

### 6.1 Recommendations for IoB-driven health coaching systems

The development and deployment of IoB-enabled health coaching systems require consideration of technical robustness, user-centred design, clinical effectiveness, and regulatory compliance. Several interrelated recommendations emerge from the comparative synthesis and quality assessment conducted in this review. The synthesized reference architecture and evaluation framework were derived from recurring architectural, technological, and methodological patterns across the included studies and should be interpreted as conceptual synthesis models intended to guide future research and system development rather than as universally validated standards.

#### 6.1.1 Technical architecture and system design

A primary recommendation is the implementation of modular and multi-layer IoT architectures that can seamlessly integrate wearable sensors, ambient monitors, cloud services, and edge intelligence (2, 49, 78). By deploying edge computing nodes, real-time event detection (e.g., falls, arrhythmia, glucose spikes) can support low-latency event detection, reducing dependency on constant cloud connectivity. Furthermore, federated learning and on-device AI models help ensure privacy-preserving analytics, which is critical for sensitive healthcare applications (79). Future systems should consider lightweight communication protocols such as MQTT alongside healthcare interoperability standards such as HL7/FHIR to support data exchange across connected devices and clinical information systems. Architectural resilience should include redundancy, failover mechanisms, and resource-aware optimization to handle the complexity of multi-modal sensor data streams.

#### 6.1.2 Behavioural intelligence and personalization

Effective health coaching relies on precise behavioural modelling that accounts for individual preferences, readiness for change, and historical health patterns. Integrating behavioural theories such as the Transtheoretical Model, Self-Determination Theory, and Cognitive Behavioural Theory may strengthen the theoretical grounding and contextual relevance of adaptive interventions (22, 23, 80). Adaptive feedback loops where the system shifts from informative to motivational prompts based on adherence levels may support engagement and behavioural adaptation, although sustained effects require longitudinal validation. Leveraging multi-modal fusion (wearable, physiological, and ambient data) enhances the accuracy of behaviour recognition, anomaly detection, and intervention timing (81, 82). Incorporating gamification, cognitive nudges, and conversational agents further strengthens personalization and user trust.

#### 6.1.3 Privacy, security, and ethical compliance

The collection of intimate behavioural and physiological data mandates rigorous attention to privacy and security. IoB systems should implement proportionate privacy and security safeguards, including encryption, access control, data minimization, and, where appropriate, privacy-preserving or auditable mechanisms such as federated learning or blockchain-based integrity verification (75, 83). Ethically, dynamic or granular consent mechanisms should be considered where continuous sensing and secondary behavioural inference create evolving data-use contexts, enabling users to grant, revoke, and customize permissions for data usage. Embedding explainable AI (XAI) techniques is also recommended to improve transparency and trustworthiness in algorithmic decision-making for health interventions (84). Compliance with GDPR, HIPAA, and local health data regulations ensures cross-jurisdictional acceptability and reduces liability.

#### 6.1.4 Clinical validation and user-centric evaluation

The clinical effectiveness of IoB-driven health coaching must be validated using rigorous experimental designs, including randomized controlled trials, longitudinal pilot studies, and cross-population validation (14, 39). Outcome metrics should include both physiological (e.g., blood pressure, glucose levels, sleep quality) and behavioural (e.g., adherence rates, engagement duration) indicators. Equally important is user experience evaluation, which influences long-term adoption. Collecting qualitative and quantitative feedback through mobile app usage analytics, surveys, and focus groups provides insights into trust, usability, and sustained motivation (85, 86). Future evaluations should integrate technical performance with behavioural, user-centred, implementation, and clinically meaningful outcomes rather than relying predominantly on predictive accuracy.

#### 6.1.5 Policy, scalability, and future directions

To transition IoB systems from experimental to mainstream healthcare, policy frameworks must support standardization, funding incentives, and ethical oversight (87, 88). Governments and healthcare institutions could support carefully governed pilot evaluations in comparatively established application areas, including elderly care, remote patient monitoring, and chronic disease management, while requiring appropriate clinical, ethical, and implementation oversight (19, 89). Future research should prioritize the integration and rigorous validation of digital twins and immersive health simulations for personalized risk modelling (56, 90, 91, 92) scalable federated learning ecosystems for privacy-conscious cross-institutional model development (93), and emotion- and context-aware interventions using LLMs and multimodal AI (22, 94). Importantly, these emerging directions should be evaluated through real-world, longitudinal, and clinically relevant studies rather than technical feasibility alone.

### 6.2 Final synthesis

In conclusion, the findings of this review indicates that IoB-enabled adaptive health coaching has established a credible foundation in continuous sensing, behavioural and physiological inference, remote monitoring, and selected chronic disease and elderly-care applications. However, evidence of sustained behaviour change, long-term clinical effectiveness, and large-scale implementation remains less consistent, particularly for advanced adaptive interventions, digital twins, behavioural simulation, and emerging AI- or LLM-enabled systems. The synthesized reference architecture and evaluation framework developed in this review integrate recurring architectural components, intervention mechanisms, and evaluation criteria identified across the included studies; they should therefore be interpreted as evidence-informed conceptual synthesis models rather than universally validated standards. Future progress will depend on longitudinal and multi-site validation, representative datasets, reproducible system reporting, explicit integration of behavioural theory, interoperable clinical workflows, and robust privacy, fairness, and governance mechanisms. Addressing these priorities is essential for translating IoB from technically promising monitoring and prediction systems into clinically meaningful, ethically responsible, and scalable adaptive health coaching ecosystems.

## Data Availability

The original contributions presented in the study are included in the article/Supplementary Material, further inquiries can be directed to the corresponding author.
