# Supplementary material for: A systematic review and evaluation framework for IoB-based adaptive health coaching systems
Source: Front Digit Health. 2026 Aug 21;8:1816418. doi: 10.3389/fdgth.2026.1816418 (PMC13543663; doi:10.3389/fdgth.2026.1816418)
Supplement: Supplementary file 1 [file Table1.docx]

**Supplementary Table S1.** Database-Specific Search Strategies, Search Fields, Filters, and Retrieval Results

| **Database** | **Search Strategy**  **(Exact Database Syntax)** | **Search Fields & Filters Applied** | **Limits / Restrictions** | **Search Date** | **Records Retrieved** |
| --- | --- | --- | --- | --- | --- |
| IEEE Xplore | (("All Metadata":"Internet of Behaviours" OR "All Metadata":IoB) AND ("All Metadata":"health coaching" OR "All Metadata":"adaptive intervention" OR "All Metadata":"personalized intervention" OR "All Metadata":"Just-in-Time Adaptive Intervention" OR "All Metadata":JITAI OR "All Metadata":"behavioural analytics") AND ("All Metadata":"IoT healthcare" OR "All Metadata":wearables OR "All Metadata":"digital health" OR "All Metadata":"remote patient monitoring")) | Fields: All Metadata (Title, Abstract, Index, Keywords) | Language: English    Pub Type: Journals, Conferences    Dates: Jan 2015 – Dec 2025 | 15 Dec 2025 | 68 |
| Scopus | TITLE-ABS-KEY ( ( "Internet of Behaviours" OR "IoB" ) AND ( "health coaching" OR "adaptive intervention" OR "personalized intervention" OR "Just-in-Time Adaptive Intervention" OR "JITAI" OR "behavioural analytics" ) AND ( "IoT healthcare" OR "wearables" OR "digital health" OR "remote patient monitoring" ) ) AND ( LIMIT-TO ( LANGUAGE , "English" ) ) AND ( LIMIT-TO ( DOCTYPE , "ar" ) OR LIMIT-TO ( DOCTYPE , "cp" ) ) | Fields: Title, Abstract, Keywords (TITLE-ABS-KEY) | Language: English    Pub Type: Article (ar), Conference Paper (cp)    Dates: 2015 – 2025 | 15 Dec 2025 | 142 |
| Web of Science (Core Collection) | TS=(("Internet of Behaviours" OR "IoB") AND ("health coaching" OR "adaptive intervention" OR "personalized intervention" OR "Just-in-Time Adaptive Intervention" OR "JITAI" OR "behavioural analytics") AND ("IoT healthcare" OR "wearables" OR "digital health" OR "remote patient monitoring")) | Fields: Topic (TS searches Title, Abstract, Keywords) | Language: English    Pub Type: Articles, Proceedings Papers    Dates: 2015-01-01 to 2025-12-15 | 15 Dec 2025 | 106 |
| PubMed | (("Internet of Behaviours"[TIAB] OR "IoB"[TIAB]) AND ("health coaching"[TIAB] OR "adaptive intervention"[TIAB] OR "personalized intervention"[TIAB] OR "Just-in-Time Adaptive Intervention"[TIAB] OR "JITAI"[TIAB] OR "behavioural analytics"[TIAB]) AND ("IoT healthcare"[TIAB] OR "wearables"[TIAB] OR "digital health"[TIAB] OR "remote patient monitoring"[TIAB])) OR (("Internet of Things"[Mesh] OR "Monitoring, Ambulatory"[Mesh]) AND "Behavior Therapy"[Mesh] AND "Telemedicine"[Mesh]) | Fields: Title/Abstract ([TIAB]) and Medical Subject Headings ([Mesh]) | Language: English    Pub Type: Journal Article    Dates: 2015/01/01 to 2025/12/15 | 15 Dec 2025 | 64 |
| ACM Digital Library | (acmdlTitle:("Internet of Behaviours" "IoB") OR abs:("Internet of Behaviours" "IoB") OR keyword:("Internet of Behaviours" "IoB")) AND (acmdlTitle:("health coaching" "adaptive intervention" "personalized intervention" "Just-in-Time Adaptive Intervention" "JITAI" "behavioural analytics") OR abs:("health coaching" "adaptive intervention" "personalized intervention" "Just-in-Time Adaptive Intervention" "JITAI" "behavioural analytics") OR keyword:("health coaching" "adaptive intervention" "personalized intervention" "Just-in-Time Adaptive Intervention" "JITAI" "behavioural analytics")) AND (acmdlTitle:("IoT healthcare" wearables "digital health" "remote patient monitoring") OR abs:("IoT healthcare" wearables "digital health" "remote patient monitoring") OR keyword:("IoT healthcare" wearables "digital health" "remote patient monitoring")) | Fields: Expanded Title, Abstract, Author Keywords | Language: English    Pub Type: Research Articles, Conference Proceedings    Dates: Jan 2015 – Dec 2025 | 15 Dec 2025 | 32 |
| Total Across Databases |  |  |  |  | 412 |
| Duplicates Removed | *Automated removal using EndNote 20 & manual verification.* |  |  |  | - 56 |
| Unique Records Screened |  |  |  |  | 356 |

Note: ACM = Association for Computing Machinery; IEEE = Institute of Electrical and Electronics Engineers; JITAI = Just-in-Time Adaptive Intervention; MeSH = Medical Subject Headings; PRISMA = Preferred Reporting Items for Systematic Reviews and Meta-Analyses; TIAB = Title/Abstract; TS = Topic Search; WoS = Web of Science.
