# Supplementary material for: A systematic review and evaluation framework for IoB-based adaptive health coaching systems
Source: Front Digit Health. 2026 Aug 21;8:1816418. doi: 10.3389/fdgth.2026.1816418 (PMC13543663; doi:10.3389/fdgth.2026.1816418)
Supplement: Supplementary file 2 [file Table2.docx]

**Supplementary Table S2:** Synthesis of the 75 Included Empirical Peer-Reviewed Studies

| **Reference No** | **Study** | **Publication Type** | **Study Design** | **Healthcare Domain** | **Population / Dataset** | **IoB/IoT Components** | **Methods** | **Evaluation Setting** | **Key Outcomes** |
| --- | --- | --- | --- | --- | --- | --- | --- | --- | --- |
| **(15)** | Ali et al. (2025) | Journal | Experimental | Remote Patient Monitoring | Synthetic & Clinical Body Sensor Data | Human Body Communication (HBC) transceivers, on-body nodes | Robust Auto-Encoder, Conditional GAN (CGAN) channel modelling | Simulation & Laboratory Testbed | Achieved precise, low-power digital signal transmission through human tissue with high signal-to-noise ratio. |
| **(19)** | Amiri et al. (2023) | Journal | Review / Mapping | Personal Digital Health and IoB Applications | Published literature on ML-enabled IoB healthcare applications | IoB ecosystem, IoT devices, wearable sensors, mobile health applications | Machine Learning (ML), Deep Learning (DL), behavioural analytics, taxonomy development | Literature-based synthesis | Successfully categorized personal health applications into behavioural risk tiers, optimizing feedback loops. |
| **(37)** | Loughnane et al., (2025) | Journal | Systematic Review | Mental Health Support | Published literature on digital health coaching interventions | Digital health platforms, mobile health (mHealth), wearable technologies, AI-enabled coaching systems | Hybrid human-AI interaction models, Human coaching, AI-driven health coaching, | Literature-based synthesis | Compared human, AI, and hybrid health coaching approaches in digital health interventions |
| **(41)** | Sqalli, M. T. & Al-Thani, D., (2019) | Conference | System architecture and prototype study | Chronic disease management | Patients with chronic diseases (conceptual healthcare model) | Mobile health platform, wearable devices, IoT sensors, cloud-based health monitoring | Artificial Intelligence, decision-support algorithms, personalized health coaching | Prototype architecture with conceptual evaluation | Proposed an AI-supported health coaching framework that integrates IoT-based health monitoring and personalized decision support to facilitate continuous chronic disease management and improve patient self-management. |
| **(46)** | Bobhate & Bhaladhare (2025) | Conference | Experimental study | Mental health (Depression detection) | Depression-related behavioural dataset | Internet of Behavior (IoB), digital behavioural data | Deep Learning for depression detection | Experimental evaluation | Investigated the use of deep learning models within an IoB framework for depression detection, demonstrating the potential of behavioural data |
| **(21)** | Chen et al. (2024) | Journal | Quasi-Experimental | Chronic Kidney Disease (CKD) Care | Pre-dialysis CKD patient cohorts | Interactive conversational robot, home health gateways | Virtual Health Coaching analytics, rule-based clinical NLP engines | Quasi-experimental clinical evaluation | Substantially enhanced patient health literacy and treatment adherence regarding strict fluid restriction targets. |
| **(2)** | Chien et al. (2025) | Journal | Content & Bibliometric Analysis | Long-Term Care for Older People | Longitudinal health datasets of aging citizens | Edge intelligence units, ambient room sensors, indoor gateways | Deep Learning-driven anomaly classification, edge analytics optimization | Long-Term Institutional Eldercare Facilities | Documented critical performance improvements in processing low-latency emergency alerts (e.g., falls) using edge nodes. |
| **(67)** | Abdollahi et al. (2022) | Journal | Experimental | Eldercare & Mental health | Older adults living in a senior care facility | Socially assistive robot with multimodal sensing | Artificial emotional intelligence, emotion recognition, human–robot interaction | Pilot study with older adults | The empathic AI-enabled robot improved user engagement, mood. |
| **(65)** | De Caro & Zimeo (2024) | Journal | Software Architecture Validation | Ambient Health & Well-Being | Renewable Energy Community user profiles | Smart home IoT meters, environmental sensors | Self-adapting behavioural state machines, predictive usage profiles | Field Deployment Testbed | Successfully balanced user thermal comfort preferences with clean energy generation targets. |
| **(23)** | Del-Valle-Soto et al. (2024) | Journal | Comprehensive Review / Mapping | Health & Well-Being | Consumer wearable user tracking logs | Multi-brand commercial smartwatches, motion trackers | Behaviour Change Technique (BCT) classification frameworks | Cross-Platform Integration Testbed | Identified and mapped specific BCT clusters that successfully maximize long-term active lifestyle compliance. |
| **(85)** | Parween et al. (2025)) | Journal | Experimental system development | Human movement monitoring and digital health | Human movement monitoring data | IoT sensors, Digital Twin platform | Digital Twin modelling, IoT data integration | Prototype implementation and experimental evaluation | Developed a digital twin-enabled IoT framework for human. |
| **(83)** | Shalu et al. (2024) | Conference | Experimental system development | Intelligent healthcare and disease diagnosis | Human healthcare data (IoB-enabled intelligent healthcare framework) | Internet of Behaviours (IoB), IoT sensors, connected healthcare devices | Artificial Intelligence–based disease diagnosis and behavioural data analytics | Experimental system evaluation | Proposed and evaluated an IoB-based intelligent healthcare framework. |
| **(52)** | Ilukpitiya et al. (2024) | Conference | Empirical Evaluation | Fitness Coaching & Wellness | Users performing personalized fitness and nutrition programs | Wearable sensors, real-time exercise monitoring, personalized fitness platform | Artificial Intelligence, body type classification, personalized workout and nutrition recommendation | Prototype implementation | Developed an AI-driven personalized fitness coaching system. |
| **(82)** | Goel et al. (2023) | Journal | Experimental study | Chronic Disease Management | Real-time Medical IoT data | Medical IoT devices, distributed healthcare systems | Federated Learning (FL), secure multi-party computation algorithms | Experimental evaluation | Proposed and evaluated a federated learning framework for real-time Medical IoT that improves privacy while maintaining predictive accuracy for chronic disease monitoring. |
| **(88)** | Gu et al. (2025) | Journal | Case study | Elderly care infrastructure | Big data related to elderly care facilities | Big data analytics for spatial decision-making | Natural Language Processing (NLP), rule engines | Case study | Proposed a data-driven framework for optimizing the location of elderly care facilities. |
| **(10)** | Heidari et al. (2025) | Journal | System Validation Study | Multi-user Cloud Healthcare | Distributed multi-user clinical telemetry logs | Blockchain nodes, edge routers, biological data forwarders | Deep Reinforcement Learning (DRL), secure cryptographic offloading | Simulated Multi-user Cloud Infrastructure | Dramatically minimized data offloading processing delay times while maintaining zero data-tampering vulnerabilities. |
| **(62)** | Hou & Liu (2025) | Journal | Predictive Modeling | Dementia Awareness & Memory Care | Community adult learners | Interactive web logs, cognitive assessment toolkits | Artificial intelligence classifiers, spatial environment analyzers | Community Educational Setting | Mapped environmental attributes that directly correlate with higher dementia care safety and resident orientation. |
| **(28)** | Hu et al. (2025) | Journal | Experimental Architecture | Industrial Health & Safety | Factory workers monitoring logs | Industrial wearable wristbands, environment beacons | Federated Learning with selective state space models (SSMs) | Industrial Plant Prototype Deployment | Delivered highly lightweight, privacy-preserving worker exhaustion and hazardous movement recognition. |
| **(76)** | Hwang et al. (2025) | Journal | Scoping Review / Evaluation | Adaptive Health Coaching | Preventive care user cohorts | Gamified mobile apps, wearable fitness accessories | Behavioral reward mapping, adaptive feedback algorithms | Digital Wellness Platform Evaluation | Defined optimal gaming feedback cycles that prevent user notification burnout and encourage long-term interaction. |
| **(8)** | Ioannidou et al. (2025) | Journal | Framework Validation | Assistive Healthcare Robotics | Multi-device assistive care spaces | Humanoid robots, clinical EHR portals, smart room nodes | Healthcare Robotics' Ontology (HERON) logic, semantic graphs | Controlled Geriatric Care Unit | Achieved seamless, error-free automated communication across multi-vendor clinical devices and moving robots. |
| **(50)** | Karahoca et al. (2017) | Journal | Empirical Evaluation | Institutional Healthcare IT | Digital health adoption workflows | IoT-enabled healthcare technologies | Technology Acceptance Model (TAM), behavioural intention analysis | Survey-based empirical evaluation | Examined the factors influencing users' intention to adopt IoT-based healthcare technologies.. |
| **(7)** | Ji et al. (2025) | Journal | Pilot Deployment | Networked Elderly Care | Semi-independent aging individuals | Nursing robots, indoor camera grids, wearable vital patches | Intelligent networked robotics, multi-modal sensor fusion | Smart Assisted-Living Facility | Achieved safe 24/7 autonomous monitoring of vulnerable seniors, drastically speeding up emergency response times. |
| **(81)** | Sankar & Usha (2024) | Journal | System development and experimental evaluation | Rural healthcare and patient monitoring | Health coaching app user base | healthcare IoT environment | Fuzzy intelligence system, blockchain-based secure patient monitoring and record management | Prototype implementation and experimental evaluation | Proposed a blockchain-enabled IoB framework integrated with fuzzy intelligence for continuous patient monitoring |
| **(51)** | Jörke et al. (2024) | Journal | Systematic Review | Motivational Coaching | Conversational coaching database | Voice assistants, mobile chat applications | Conversational agents, Motivational Interviewing (MI) rule sets | Consolidated Interaction Database | Documented that automated empathetic conversational styles yield significantly higher health adherence rates. |
| **(75)** | Javaid et al. (2022) | Journal | Review article | IoB-enabled healthcare systems | Published literature | Internet of Behaviours (IoB), IoT-enabled healthcare technologies | Behavioural analytics, AI-enabled healthcare systems | Literature review | Reviewed the key features, enabling technologies, facilitators, challenges, and future opportunitie. |
| **(40)** | Kumar et al. (2023) | Journal | Trend Analysis / Review | Healthcare IoT Foundations | Multi-source telehealth infrastructures | Clinical wireless nodes, smart e-health gateways | Healthcare IoT (H-IoT) trends, cryptographic security layers | Simulated Hospital Testbed | Established baseline communication standards required to securely integrate telemetry records with IoB frameworks. |
| **(47)** | Zhang & Di (2025) | Journal | Cross-sectional empirical survey | Elderly care and healthy aging | Older adults living alone in Shaanxi, China | IoB/IoT component | Kano model, ERG theory, multivariate ordinal logistic regression | Community-based survey in Shaanxi, China | Identified medical and mental health services as priority needs and key factors influencing elderly care demand. |
| **(89)** | Kim et al. (2024) | Journal | Cross-sectional empirical survey | Digital healthcare device adoption | Adult users of healthcare devices across different age groups | Smart healthcare devices | Technology Acceptance Model (TAM), behavioural intention analysis | Questionnaire-based empirical evaluation | Identified device type and generation as key factors influencing healthcare device acceptance and adoption. |
| **(90)** | Lin et al. (2025) | Journal | Analytical Evaluation | Home Healthcare Robotics | Elderly home care patients and providers | Home care assistant robots, vitals check modules | Unified Theory of Acceptance (UTAUT), Fuzzy AHP methods | Home Care Stakeholder Analysis | Structured key operational safety parameters required for successful autonomous home health assistant rollouts. |
| **(20)** | Liu et al. (2025) | Journal | System Evaluation | Smart Elderly Care Institutions | Institutionalized elderly populations | Smart institutional beds, motion trackers, gateway dashboards | Synergistic service-technology evaluation metrics | Institutional Field Evaluation | Proven that combining automated sensor updates with regular human caregiver oversight minimizes staff fatigue. |
| **(61)** | Loebel & Gittens (2025) | Conference | Conceptual study | Psychosocial health of older adults | Older adult | Social robots, digital companions | E Human–robot interaction | Conceptual analysis | Explored the potential of social robots to support psychosocial well-being in older adults |
| **(6)** | Lu et al. (2025) | Journal | Algorithmic Evaluation | Automated Insulin Delivery | Type 1 Diabetes patient cohorts | Continuous glucose sensors, smart insulin pump actuators | Event-triggered Adaptive Dynamic Programming (ADP) | Clinical Simulation Testbed | Enabled automated, personalized meal-bolus delivery adjustments that effectively prevent overnight hypoglycemia. |
| **(44)** | Damaševičius et al. (2023) | Conference | Experimental | Adaptive Lifestyle Coaching | Obesity and sedentary risk cohorts | Internet of Behaviours | Behavioural decision support | Conceptual analysis | Discussed IoB concepts, behavioural decision-making, and future implications across digital environments. |
| **(64)** | Majid Al-Taee et al. (2025) | Conference | Prototype Testing | Juvenile Diabetes Management | Pediatric Type 1 Diabetes cohorts | Humanoid robot coaches, blood glucose meters, kid smartwatches | IoT platform robot-child interaction models, rule engines | Pediatric Clinic Pilot | Increased young patients' long-term testing compliance and dietary understanding through interactive play. |
| **(33)** | Mane et al. (2025) | Journal | Experimental Validation | Emergency Elderly Monitoring | Isolated seniors living at home | Non-wearable ambient motion detectors, acoustic arrays | Non-responsive state detection, threshold anomaly logic | Single-Resident Home Deployment | Dispatched immediate relative notifications when unexpected, long-duration room immobility trends occurred. |
| **(91)** | Mishra et al. (2025) | Journal | Experimental Validation | Environment-Aware Telehealth | Respiratory health patient test spaces | Gas detection nodes, ambient noise microphones | Deep Learning, environmental audio classification networks | Controlled Lab Environment | Identified and flagged hazardous indoor air particulate spikes likely to induce severe asthma triggers. |
| **(79)** | Montagna et al. (2025) | Journal | System development and experimental evaluation | Hypertension self-management | Hypertensive patients | Digital health platform, patient self-management system | Privacy-preserving Large Language Model (LLM)-based chatbot | Prototype implementation and evaluation | Developed a privacy-preserving LLM chatbot that supported personalized hypertension self-management while protecting patient data.. |
| **(29)** | Motger et al. (2025) | Journal | Algorithmic Validation | Mobile App Health Optimization | Public health mobile application users | Mobile application user feedback repositories | Encoder-only Large Language Models, automated feature extractions | Software Optimization Benchmarks | Streamlined health application feature updates by extracting user performance bugs automatically. |
| **(92)** | Pace et al. (2019) | Journal | System architecture and experimental evaluation | Remote healthcare monitoring | Body Sensor Network (BSN) data | IoT, edge computing, cloud computing, wearable sensors | Edge-based IoB routing architectures, stream priority scheduling | Prototype implementation and performance evaluation | roposed an edge-based healthcare architecture that improved real-time processing, reduced latency, and enhanced efficiency for IoT-enabled health monitoring applications.. |
| **(19)** | Amiri et al. (2023) | Journal | Evaluation Study | Secure Health Monitoring IoT | Connected healthcare facility systems | Encrypted physiological sensor streams, security keys | AI-powered security auditing, anomaly isolation models | Vulnerability Testing Network | Deflected malicious network spoofing attempts aimed at corrupting continuous patient vitals streams. |
| **(93)** | Saravanakumar et al. (2022) | Journal | Theoretical & Empirical Evaluation | Lifelogging Health Ecosystems | Chronic disease patient self-trackers | Automated lifelogging cameras, passive context logs | Multi-modal lifelogging data analytics, risk/reward assessment | Institutional Lab Evaluation | Balanced the immense data benefits of detailed lifestyle tracking with necessary user privacy guidelines. |
| **(14)** | Ogundele et al. (2025) | Journal | Validation Study | Eldercare Health Monitoring | Elderly home care patients, clinical remote teams | Home activity logging systems, linguistic alert synthesis tools | Automated natural language summary generators, semantic mapping | Remote Clinical Care Center | Translated messy raw multi-sensor activity graphs into clean text updates, lowering clinical review fatigue. |
| **(101)** | Nitin et al. (2025) | Conference | Experimental study | IoT healthcare monitoring | Real-time healthcare IoT device data | IoT healthcare devices, real-time sensor monitoring | Long Short-Term Memory (LSTM) for anomaly detection | Experimental performance evaluation | Developed an LSTM-based framework for real-time anomaly detection in IoT healthcare devices. |
| **(69)** | Österholm et al. (2025) | Journal | Qualitative Validation | Welfare Technology Implementation | Municipal elder care decision makers | Municipal health portal interfaces | Qualitative semantic analysis, implementation readiness mapping | Swedish Municipal Elder Care Units | Provided clear operational implementation metrics for safely introducing welfare technology across nursing homes. |
| **(94)** | Akkaş et al. (2020) | Journal | System development and experimental evaluation | Remote patient monitoring | IoT-enabled patient monitoring system | IoT sensors, wearable devices, cloud-based health monitoring | Real-time physiological data acquisition and monitoring | Prototype implementation and performance evaluation | Developed an IoT-based patient monitoring system for real-time remote healthcare monitoring. |
| **(95)** | L.Zhang et al. (2025) | Journal | Algorithmic Evaluation | Wearable Health Monitoring & Smart Clothing | Multi-posture physiological tracking profiles | Intelligent fabric sensors, on-body hub node coordinators, Human Body Channel (HBC) transceivers | Proximal Policy Optimization (PPO), deep reinforcement learning | High-throughput multi-posture network simulator | Improved communication efficiency and network performance through dynamic hub-position optimization in body-centric IoB networks. |
| **(96)** | Jahn et al. (2025) | Conference | International multi-site project evaluation | Active and healthy aging; digital health coaching for older adults | Community-dwelling older adults across European and Japanese e-VITA settings | Digital coaching devices, social robots, connected digital platform, smart-home | LLM-based conversational coaching, personalized digital interaction, | Real-world international e-VITA project settings in Europe and Japan | Identified culturally distinct uses of LLM-based health coaching devices, highlighting the need for personalized |
| **(30)** | Pawana et al. (2025) | Journal | Algorithmic Evaluation | Diabetes Management Control | Insulin pump users | Connected IoT medical devices (IoTMD), wireless pump links | Lightweight LLM anomaly detection framework | Simulated Medical Network | Thwarted malicious sensor-tampering attacks meant to deliver improper, dangerous insulin dosages. |
| **(38)** | Rahmani et al. (2018) | Journal | System Evaluation Study | Edge-Cloud Healthcare | Multi-sensor home monitoring patients | Smart e-health gateways, edge switches, vital sensors | Fog computing data orchestration, decentralized filtering | Real-world Edge Testbed Field Deployment | Demonstrated massive reductions in internet traffic data volume by pre-filtering raw sensor signals at the edge. |
| **(3)** | Rehman et al. (2025) | Journal | Trend & Bibliometric Study | Global Healthcare IoT Research | Multi-decade international health records | Medical IoT hardware nodes, communication systems | Longitudinal trend analytics, cross-vendor innovation mapping | Global Health Registry Database | Documented global medical IoT adoption rates while establishing standardized safety guidelines. |
| **(97)** | Reichenpfader et al. (2024) | Journal | Scoping Review / Evaluation | Radiology Information Extraction | Multi-hospital medical imaging databases | Radiology text servers, electronic medical record banks | LLM-based information extraction, named entity recognition | Clinical Document Validation | Automated the extraction of vital cancer tracking findings from unstructured clinical texts with high precision. |
| **(26)** | Rimsha Jamil Ghilzai et al. (2025) | Journal | Algorithmic Evaluation | Heart Disease Prediction | Cardiology outpatients | Digital ECG capture systems, wearable heart monitors | Deep Learning-based ECG image analysis, custom CNNs | Clinical Outpatient Dataset | Achieved high classification accuracy in identifying complex cardiac arrhythmias from basic pixel readouts. |
| **(22)** | Ristianto et al. (2025) | Journal | Systematic Literature Review | Student Health & Education | Digital education student cohorts | Educational gamification applications, mobile interfaces | Personalized gamification design, user performance clustering | Academic Digital Environment | Proved that personal activity milestones prevent wellness program dropouts among younger student demographics. |
| **(55)** | Mahapatra et al. (2025) | Journal | System development and experimental performance evaluation | Predictive health monitoring and remote patient monitoring | Physiological health data collected/processed through WBAN-based monitoring scenarios | Wireless Body Area Network (WBAN), body sensors, Digital Twin, connected health monitoring | Machine learning-based predictive health analytics integrated with Digital Twin modelling | Experimental evaluation of the proposed WBAN–Digital Twin healthcare framework | Integrated WBAN, Digital Twin, and machine learning to enable predictive health monitoring and early identification of health risks. |
| **(73)** | Saba et al. (2025) | Journal | Simulation Study | EV Energy Infrastructure Care | Connected smart hospital fleet vehicles | Vehicle-to-Grid (V2G) battery meters, charging nodes | Twin-Delayed Deep Deterministic Policy Gradient (TD3) | Smart City Infrastructure Grid | Optimized vehicle power allocation to ensure uninterrupted emergency operations during hospital blackouts. |
| **(63)** | Sayabalian & Kunze (2025) | Journal | Empirical Commentary / Evaluation | Memory Care Facilities | Assisted living residents with dementia | Wall-mounted ambient sensors, personal location tags | Exposure risk mitigation modeling, behavioral vulnerability tracking | Assisted Living Memory Unit | Established critical safety balances to track wandering residents without infringing on human rights. |
| **(71)** | Schmidt & Higgins (2025) | Journal | Systematic Methodology | Systematic Behaviour Data Extraction | IoB research literature databases | Systematic review software interfaces, text mining nodes | Automated systematic data extraction frameworks | Multi-study Review System | Validated text-mining code scripts that help researchers isolate valid healthcare findings from messy raw papers. |
| **(5)** | Shaikh (2025) | Conference | Framework Review / Mapping | Smart Healthcare Systems | Internet of Medical Things (IoMT) users | Embedded medical tracking modules, wireless transceivers | IoMT analytical workflows, edge communication protocols | Simulated Health Ecosystem | Structured the exact protocol bridges needed to securely connect personal fitness bands with hospital servers. |
| **(58)** | Sharma et al. (2025) | Journal | Clinical Validation | Chronic Remote Monitoring | Patients with advanced COPD | Connected pulse oximeters, smart spirometers | Machine Learning, multi-sensor remote patient monitoring | Ambulatory Outpatient Care Trial | Significantly reduced clinical emergency readmissions by tracking chronic symptoms proactively. |
| **(25)** | Sharma et al. (2025) | Journal | Empirical Validation | COPD Nutrition & Care Optimization | Severe COPD patient cohorts | Nutritional tracking portals, wearable exercise sensors | Personalized nutrition algorithms, biometric sensor monitoring | Specialized Clinical Trial | Successfully mitigated muscle wasting risks in COPD patients through automated, tailored dietary feedback. |
| **(98)** | Mohan et al. (2025) | Journal | Cooperative AI model development and experimental evaluation | Older-adult fall-risk prediction and preventive care | Public repository data combining vital signs and ADL information | Continuous vital-sign monitoring, wearable 3D accelerometer data, ADL monitoring | Fuzzy Logic for vital signs; Deep Belief Network (DBN) for ADL patterns; Random Forest meta-model | Offline experimental evaluation against the Morse Fall Scale (MFS) | Achieved 90.0% accuracy, 85.71% sensitivity, and 100% specificity for proactive fall-risk prediction in older adults. |
| **(99)** | Taif Ghiwaa et al. (2024) | Conference | Framework Testing | Telemedicine System Adoption | Multi-regional digital clinic test cases | On-body medical telemetry sensors, home telehealth hubs | Internet of Body-based Telemedicine Adoption Framework (IoB-TMAF) | Regional Telemedicine Network | Isolated the technical usability factors needed to boost tele-consultation adoption rates in rural zones. |
| **(100)** | Woznowski et al. (2015) | Conference | System architecture development and prototype evaluation | Residential healthcare | Multimodal residential sensor data | Wearable and environmental sensors, IoT | Multimodal activity and behaviour monitoring | Smart-prototype | Enabled continuous health and behaviour monitoring using multimodal sensors. |
| **(12)** | Vultureanu-Albişi et al. (2025) | Journal | Framework Validation | Next Generation-IoT Explainability | Complex multi-sensor environment nodes | Distributed edge computing units, notification displays | eXING-IoT conceptual explainability integration frameworks | Complex Simulation Testbed | Enabled automated systems to output clear explanations describing exactly why a specific health alert was generated. |
| **(31)** | Wang & Yan (2024) | Journal | Observational Study | China’s Elderly Mutual Aid | Local senior citizen mutual care networks | Mutual aid mobile coordination logs, help buttons | Active aging perspective analysis, service demand mapping | Field Community Evaluation | Provided operational metrics on how simple mobile alerts can optimize community-based elder care check-ins. |
| **(53)** | Oleribe et al. (2025) | Journal | ethical analysis | AI-enabled healthcare Healthcare IoT | Healthcare AI literature | AI-driven digital health system | Ethical AI analysis | Institutional Health System | Identified ethical challenges and priorities for responsible AI adoption in healthcare. |
| **(59)** | Lu et al. (2019) | Journal | Randomized controlled trial | Hypertension management | Adults with hypertension | CInteractive mHealth system, mobile monitoring | Personalized behavioural feedback and self-management support | Clinical intervention | Improved blood pressure control through interactive mobile health intervention. |
| **(54)** | Sruthi Sai Prabha et al. (2025) | Conference | System development and evaluation | Personalized eldercare | Senior home care single residents | Connected eldercare system, local edge devices | Local LLMs, multi-agent AI, personalized reasoning | Prototype evaluation | Developed a local LLM and multi-agent framework for personalized, privacy-aware eldercare support. |
| **(86)** | Alawadi et al. (2021) | Conference | Platform development and evaluation Evaluation | Federated Healthcare IoT | IoT health-monitoring data | IoT sensors, connected health platform | Federated interactive learning | Multi-site Healthcare Network | Developed a federated IoT health platform enabling privacy-aware distributed monitoring and learning. |
| **(80)** | Alghamdi et al. (2025) | Journal | Framework development and data-driven analysis | Adaptive Health Interventions | Healthcare AI literature/data | Digital health and AI systems | XAI, interpretable ML, FIXAIH framework | Framework-based evaluation | Proposed the FIXAIH framework to improve explainability, interpretability, and trust in healthcare AI. |
| **(4)** | Yang et al. (2024) | Journal | Technical Survey / Evaluation | Intelligent Wearable Systems | Professional athletes and physical rehabilitation cohorts | Next-gen smart fabrics, stretchable strain monitors, biometric clips | Multimodal biomechanical analytics, physical fatigue profiling | Human Performance Research Laboratory | Advanced the design parameters required for washing-resilient smart garments capable of capturing clean vitals data. |
| **(78)** | Al-Shqeerat et al. (2025) | Journal | Framework development and experimental evaluation | Developmental disability detection and intervention | Multimodal speech and behavioural data | Multimodal behavioural and health data acquisition | BioNeuroFusionNet, enhanced speech analysis, behavioural analysis, multimodal AI | Experimental model evaluation | Developed a multimodal AI framework integrating speech and behavioural analysis for developmental disability detection and personalized intervention. |
| **(87)** | Alipour & Moghaddam (2025) | Conference | Framework development and evaluation | Adaptive behaviour-aware applications | Affective and behavioural data | IoB, connected sensing, adaptive feedback | Affective behaviour analysis, ABDA-IoB adaptation | Experimental evaluation | Developed an affective behaviour-driven IoB framework for personalized and context-aware adaptation. |
| **(24)** | Yu et al. (2025) | Journal | Algorithmic Validation | Automated Insulin Delivery | Dynamic insulin-dependent patient simulators | Continuous subcutaneous glucose monitors, automated pump lines | Deep Reinforcement Learning (DRL) glycemic control models | FDA-Accepted Glycemic Safety Simulator | Demonstrated superior glucose stabilization performance compared to traditional static carbohydrate formulas. |
| **(48)** | Zhou et al. (2025 | Journal | Comparative Evaluation | Community-based older adult care | Older adults and care-service data in Chongqing, China | Digital/spatial service data; no direct IoB system | Supply–demand and spatial analysis | Chongqing community care setting | Identified spatial mismatches between older adults’ care needs and community-based service provision. |
