# Supplementary material for: A systematic review and evaluation framework for IoB-based adaptive health coaching systems
Source: Front Digit Health. 2026 Aug 21;8:1816418. doi: 10.3389/fdgth.2026.1816418 (PMC13543663; doi:10.3389/fdgth.2026.1816418)
Supplement: Supplementary file 3 [file Table3.docx]

**Supplementary Table S3:** Study-Level Quality and Risk-of-Bias Assessment for Included Studies

| **Reference No** | **Study** | **Methodological Transparency** | **Dataset Quality & Reliability** | **Evaluation Rigor** | **Reproducibility** | **Clinical or Real-World Validation** |
| --- | --- | --- | --- | --- | --- | --- |
| **(15)** | Ali et al. (2025) | Yes | Partial | Yes | Partial | No |
| **(19)** | Amiri et al. (2023) | Yes | Partial | Partial | No | No |
| **(37)** | Loughnane et al. (2025) | Yes | Partial | Partial | No | No |
| **(41)** | Sqalli, M. T. & Al-Thani, D. (2019) | Yes | No | Partial | Partial | No |
| **(46)** | Bobhate & Bhaladhare (2025) | Yes | Yes | Yes | Partial | No |
| **(21)** | Chen et al. (2024) | Yes | Yes | Yes | Partial | Yes |
| **(2)** | Chien et al. (2025) | Yes | Yes | Yes | Partial | Yes |
| **(67)** | Abdollahi et al. (2022) | Yes | Partial | Yes | Partial | Partial |
| **(65)** | De Caro & Zimeo (2024) | Yes | Yes | Yes | Yes | Yes |
| **(23)** | Del-Valle-Soto et al. (2024) | Yes | Partial | Yes | Partial | Partial |
| **(85)** | Parween et al. (2025) | Yes | Partial | Yes | Partial | No |
| **(83)** | Shalu et al. (2024) | Yes | Partial | Yes | Partial | No |
| **(52)** | Ilukpitiya et al. (2024) | Yes | Partial | Partial | Partial | No |
| **(82)** | Goel et al. (2023) | Yes | Yes | Yes | Yes | No |
| **(88)** | Gu et al. (2025) | Partial | Yes | Partial | Partial | Partial |
| **(10)** | Heidari et al. (2025) | Yes | Partial | Yes | Yes | No |
| **(62)** | Hou & Liu (2025) | Yes | Partial | Partial | Partial | Partial |
| **(28)** | Hu et al. (2025) | Yes | Yes | Yes | Yes | Yes |
| **(76)** | Hwang et al. (2025) | Yes | Partial | Yes | Partial | Partial |
| **(8)** | Ioannidou et al. (2025) | Yes | Partial | Yes | Partial | Yes |
| **(50)** | Karahoca et al. (2017) | Yes | Yes | Yes | Yes | Partial |
| **(7)** | Ji et al. (2025) | Yes | Yes | Yes | Partial | Yes |
| **(81)** | Sankar & Usha (2024) | Yes | Partial | Yes | Partial | No |
| **(51)** | Jörke et al. (2024) | Yes | Yes | Partial | No | No |
| **(75)** | Javaid et al. (2022) | Yes | No | No | No | No |
| **(40)** | Kumar et al. (2023) | Yes | Partial | Yes | Partial | No |
| **(47)** | Zhang & Di (2025) | Yes | Yes | Partial | Partial | Partial |
| **(89)** | Kim et al. (2024) | Yes | Yes | Yes | Yes | Partial |
| **(90)** | Lin et al. (2025) | Yes | Partial | Partial | Partial | Partial |
| **(20)** | Liu et al. (2025) | Yes | Yes | Yes | Partial | Yes |
| **(61)** | Loebel & Gittens (2025) | Partial | No | No | No | No |
| **(6)** | Lu et al. (2025) | Yes | Yes | Yes | Yes | Partial |
| **(44)** | Damaševičius et al. (2023) | Partial | No | No | No | No |
| **(64)** | Majid Al-Taee et al. (2025) | Yes | Yes | Yes | Partial | Yes |
| **(33)** | Mane et al. (2025) | Yes | Partial | Yes | Partial | Partial |
| **(91)** | Mishra et al. (2025) | Yes | Yes | Yes | Yes | No |
| **(79)** | Montagna et al. (2025) | Yes | Partial | Yes | Partial | No |
| **(29)** | Motger et al. (2025) | Yes | Yes | Yes | Yes | No |
| **(92)** | Pace et al. (2019) | Yes | Partial | Yes | Partial | No |
| **(19)** | Amiri et al. (2023) | Yes | Partial | Yes | Yes | No |
| **(93)** | Saravanakumar et al. (2022) | Yes | Partial | Partial | Partial | Partial |
| **(14)** | Ogundele et al. (2025) | Yes | Yes | Yes | Partial | Yes |
| **(101)** | Nitin et al. (2025) | Yes | Yes | Yes | Yes | No |
| **(69)** | Österholm et al. (2025) | Yes | Partial | Partial | Partial | Yes |
| **(94)** | Akkaş et al. (2020) | Yes | Partial | Yes | Partial | No |
| **(95)** | Zhang et al. (2025) | Yes | Yes | Yes | Yes | No |
| **(96)** | Jahn et al. (2025) | Yes | Yes | Yes | Partial | Yes |
| **(30)** | Pawana et al. (2025) | Yes | Partial | Yes | Yes | No |
| **(38)** | Rahmani et al. (2018) | Yes | Yes | Yes | Yes | Yes |
| **(3)** | Rehman et al. (2025) | Yes | Yes | Partial | Partial | Partial |
| **(97)** | Reichenpfader et al. (2024) | Yes | Yes | Yes | Yes | Partial |
| **(26)** | Rimsha Jamil Ghilzai et al. (2025) | Yes | Yes | Yes | Yes | Partial |
| **(22)** | Ristianto et al. (2025) | Yes | Partial | Partial | Partial | Partial |
| **(55)** | Mahapatra et al. (2025) | Yes | Yes | Yes | Yes | No |
| **(73)** | Saba et al. (2025) | Yes | Partial | Yes | Yes | No |
| **(63)** | Sayabalian & Kunze (2025) | Yes | Partial | Partial | Partial | Partial |
| **(71)** | Schmidt & Higgins (2025) | Yes | Partial | Yes | Yes | No |
| **(5)** | Shaikh (2025) | Yes | Partial | Partial | Partial | No |
| **(58)** | Sharma et al. (2025) | Yes | Yes | Yes | Yes | Yes |
| **(25)** | Sharma et al. (2025) | Yes | Yes | Yes | Yes | Yes |
| **(98)** | Mohan et al. (2025) | Yes | Yes | Yes | Yes | No |
| **(99)** | Taif Ghiwaa et al. (2024) | Yes | Partial | Yes | Partial | Partial |
| **(100)** | Woznowski et al. (2015) | Yes | Partial | Partial | Partial | No |
| **(12)** | Vultureanu-Albişi et al. (2025) | Yes | Partial | Yes | Yes | No |
| **(31)** | Wang & Yan (2024) | Yes | Partial | Partial | Partial | Partial |
| **(53)** | Oleribe et al. (2025) | Yes | No | Partial | No | No |
| **(59)** | Lu et al. (2019) | Yes | Yes | Yes | Yes | Yes |
| **(54)** | Sruthi Sai Prabha et al. (2025) | Yes | Partial | Yes | Partial | No |
| **(86)** | Alawadi et al. (2021) | Yes | Yes | Yes | Yes | Partial |
| **(80)** | Alghamdi et al. (2025) | Yes | Partial | Yes | Partial | No |
| **(4)** | Yang et al. (2024) | Yes | Yes | Yes | Yes | Partial |
| **(78)** | Al-Shqeerat et al. (2025) | Yes | Yes | Yes | Yes | No |
| **(87)** | Alipour & Moghaddam (2025) | Yes | Yes | Yes | Yes | No |
| **(24)** | Yu et set al. (2025) | Yes | Yes | Yes | Yes | Partial |
| **(48)** | Zhou et al. (2025) | Yes | Yes | Partial | Partial | Partial |
